# The pharmacophylogenetic relationships of two edible medicinal plants in the genus *Artemisia*

**DOI:** 10.3389/fpls.2022.949743

**Published:** 2022-08-18

**Authors:** Zhanhu Cui, Siqi Li, Jiayin Chang, Erhuan Zang, Qian Liu, Baochang Zhou, Chao Li, Mengzhi Li, Xianzhang Huang, Zhongyi Zhang, Minhui Li

**Affiliations:** ^1^College of Agriculture, Fujian Agriculture and Forestry University, Fuzhou, China; ^2^The First People’s Hospital of Nanyang Affiliated to Henan University, Nanyang, China; ^3^Inner Mongolia Key Laboratory of Characteristic Geoherbs Resources Protection and Utilization, Baotou Medical College, Baotou, China; ^4^College of Traditional Chinese Medicine, Inner Mongolia Medical University, Hohhot, China; ^5^Nanyang Institute of Technology, Nanyang, China; ^6^Inner Mongolia Hospital of Traditional Chinese Medicine, Hohhot, China

**Keywords:** *Artemisia argyi*, *Artemisia indica*, pharmaphylogeny, metabolomics, network pharmacology, inflammation

## Abstract

*Artemisia argyi* and *Artemisia indica* are edible medicinal plants belonging to the genus *Artemisia* in the Asteraceae. There are many similarities in their morphology, traditional curative effect, and modern pharmacological treatment. In this study, we built distribution maps of *A. argyi* and *A. indica* in China and a phylogenetic tree of common medicinal plants in Asteraceae. Then, we verified the chemical composition changes of *A. argyi* and *A. indica* via their metabolome. Traditional efficacy and modern pharmacological action were verified by network pharmacology and *in vitro* using RAW264.7 cells. The results showed that *A. argyi* and *A. indica* are widely distributed in China, and they shared pharmaphylogeny, which provides theoretical support for the mixed use of *A. argyi* and *A. indica* in most regions of China. Furthermore, there were both similarities and differences in volatile oil and flavonoid composition between *A. argyi* and *A. indica*. The network pharmacology results showed that *A. argyi* and *A. indica* had 23 common active compounds and that both had pharmacological effects on chronic gastritis (CG). Molecular docking analyses showed that quercetin, luteolin, and kaempferol have strong binding affinities with the target proteins JUN, TP53, AKT1, MAPK3, TNF, MAPK, and IL6. The cell experiment results further demonstrated that *A. argyi* and *A. indica* treat CG *via* the NOD-like receptor pathway. Based on the theory of pharmaphylogeny, this study explored the pharmaphylogeny between *A. argyi* and *A. indica* from various perspectives to provide a basis for the substitution of *A. argyi* and *A. indica*.

## Introduction

The genus *Artemisia* belongs to the family Asteraceae, which includes more than 500 species worldwide (Abad et al., 2012), among which 186 species and 44 varieties are distributed all over China.^1^ It was recorded that there are more than 20 species of *Artemisia* that have been used as medicinal plants (Editorial Board of China Bencao, 1999). *Artemisia argyi* (*A. argyi*) and *Artemisia indica* (*A. indica*) are two edible and medicinal plants (Yang et al., 2020). On the one hand, *A. argyi* and *A. indica*, as widely eaten traditional foods, and are used to make Chinese dishes such as qingtuan and dumplings. Therefore, *A. argyi* and *A. indica* are liked and accepted by most people. On the other hand, as a medicinal plant, according to the Chinese Pharmacopeia, *A. argyi* has the effects of warming the meridian, stopping bleeding, dispersing cold, and relieving pain (Chinese Pharmacopoeia Commission, 2020). It is recorded that *A. argyi* has the effect of warming the stomach (Editorial Board of China Bencao, 1999). As a medicinal plant, *A. indica* is often used instead of *A. argyi*, mixed with it, or as part of moxibustion therapy (Huang and Liu, 1999; Qin et al., 2012). People in some areas eat *A. argyi* or *A. indica* leaves to relieve abdominal pain caused by chronic gastritis (CG) (Li et al., 2018). Essential oils, flavonoids, and terpenoids have been found in *A. argyi* (Zhang et al., 2017; Chen et al., 2021), and the chemical components in *A. indica* are similar to those in *A. argyi* (Wei et al., 2009; Qin et al., 2012). In terms of pharmacology, these compounds have anti-tumor, anti-oxidation, anti-inflammatory and other effects (Chen et al., 2021). Flavonoids are the main components of *A. argyi*, and they are also the important material basis for *A. argyi* to exert pharmacological effects. The content of flavonoids is high and easy to detect. It is often used as an index component in the content determination and fingerprint study of *A. argyi* (Xue et al., 2019; Lan et al., 2020). However, there are few studies on flavonoids in *A. indica*. Therefore, the metabolite composition and the mechanism underlying the similar pharmacological effects of the two plants are worthy of further exploration. According to the theory of pharmaphylogeny, there is a certain correlation between medicinal plant affinities, chemical composition, and curative effect (pharmacological action and traditional curative effect) (Zhou et al., 2022). Therefore, due to the pharmaphylogeny between *A. argyi* and *A. indica*, we guess that their pharmacological action, chemical composition, flavor, and meridian tropism are similar.

In view of the wide potential of *A. argyi* and *A. indica* in the food and medical industries, in this study, we investigated the pharmaphylogeny between *A. argyi* and *A. indica* from multiple perspectives using metabonomics, network pharmacology, and *in vitro* experiments. We aimed to compare the geographic distribution of the two *Artemisia* species, and the flavonoid and volatile metabolites in the plants at three growth stages. Further *in vitro* cell experiments were used to reveal their pharmacological properties. The pharmacophylogenetic study of the two *Artemisia* species was preliminarily explored by the above methods, which provided. This preliminary study provides a scientific basis for the substitution of *A. argyi* and *A. indica.*

## Materials and methods

### Investigation and collection

**FIGURE 1:**
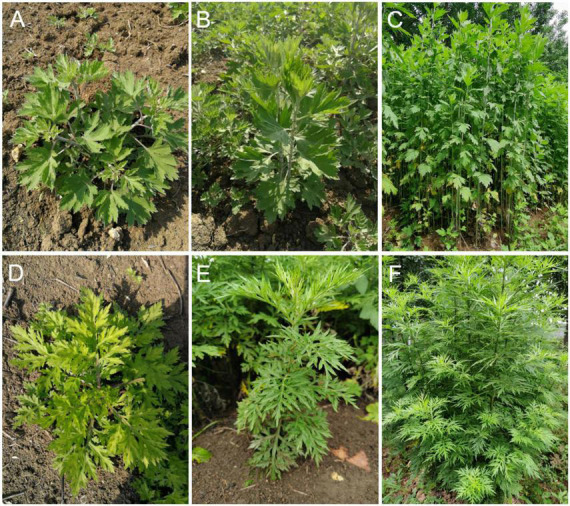
Morphological characteristics of *A. argyi* and *A. indica*. *A. argyi* **(A–C)** and *A. indica* **(D–F)** at three different growth stages (April, May, and June).

*A. argyi* and *A. indica* were collected during the growing season of 2020 from an experimental field in Nanyang County (33°03′6.56″N, 112°49′36.91″E), Henan Province, China. *A. argyi* and *A. indica* leaves were obtained at three different growth stages: the early stage (April 6, 2020), middle stage (May 16, 2020), and later stage (June 26, 2020) and were labeled ARAE, ARAM, and ARAL for *A. argyi* and ARIE, ARIM, and ARIL for *A. indica* for each growth stage, respectively. The morphological characteristics of *A. argyi* and *A. indica* at the three growth stages are shown in Figure 1.

The distribution data of *A. argyi* and *A. indica* in China were obtained from an herbarium (Chinese Virtual Herbarium),^2^ and the latitude and longitude were corrected in Google Maps and saved in a.csv format. Additionally, 19 environmental factors and 1 altitude factor, nationwide, were converted into.asc format in ArcGIS. Furthermore, 20 environmental variables, 195 points of *A. argyi*, and 133 points of *A. indica* were collected and imported into MaxEnt software for modeling analysis. The Jackknife method was used to evaluate the contribution of variables in the MaxEnt model, and the crossvalidate method was used for 10 repeated runs. The model output mode was “Logisitc,” so that the model prediction results were between 0 and 1, and the interpretation of the results was closer to the distribution probability of *A. argyi* and *A. indica*. The other parameters were default, and the output of the model was an ASCII file. The model output result file was imported into ArcGIS software and converted into a layer file in tif format. The layers were reclassified by spatial analysis tools into four levels: “unsuitable area,” “low suitable area,” “medium suitable area,” and “high suitable area,” which reflects the regional situation of *A. argyi* and *A. indica* suitable for growth in China.

### Phylogenetic tree construction

Internal transcribed spacer (ITS) gene sequences of *Artemisia* were collected for phylogenetic analysis. The ITS serves as a research region in phylogeny, biogeography, and population genetics (Liu and Christer, 2017; Ren et al., 2017). *Chrysanthemum*, *Atractylodes*, and *Cirsium* in Asteraceae were selected as outgroups as they are relatively common genera in Asteraceae and are commonly used as medicinal plants. The sequence of *Artemisia* and other Asteraceae were downloaded from GenBank for analysis. Mega11.0 was used to align the sequences, the genetic distance was calculated using the Kimura 2-parameter model, and the neighbor-joining (NJ) method was used to build a phylogenetic tree. Bootstrapping was performed with 1,000 iterations on all NJ trees to calculate the support for each branch.

### Flavonoid metabolite analysis

Freeze-dried leaves were crushed using a mixer mill for 1.5 min at 30 Hz. Then, 100 mg powder was extracted overnight at 4°C using 1.0 mL 70% aqueous methanol. The extracts were filtered and analyzed using an ultra-performance liquid chromatography-electrospray ionization tandem mass spectrometry (UPLC-ESI-MS/MS) system (UPLC SHIMADZU Nexera X2; MS, Applied Biosystems 4500 Q TRAP). The analytical conditions were as follows: UPLC column, Agilent SB-C18 (1.8 μm, 2.1 mm × 100 mm); solvent system, water (0.1% formic acid), acetonitrile (0.1% formic acid); gradient program, 95:5 v/v at 0 min, 5:95 v/v at 9.0 min, 5:95 v/v at 10.0 min, 95:5 v/v at 10.1 min, 95:5 v/v at 14 min; flow velocity, 0.35 mL/min; column oven, 40°C; injection volume, 4 μL. The effluent was connected to an ESI-triple quadrupole-linear ion trap (QTRAP)-MS. The ESI source operation parameters were as follows: ion source, turbo spray; temperature, 550°C; ion spray voltage, 5,500–4,500 V; ion source gas I, gas II, and curtain gas were set at 50, 60 and 25.0 psi, respectively. Instrument tuning and mass calibration were performed with 10 and 100 μmol/L polypropylene glycol solutions in the triple quadrupole (QQQ) and linear ion trap (LIT) modes, respectively. The declustering potential and collision energy for individual multiple reaction monitoring (MRM) transitions were determined. A specific set of MRM transitions was monitored for each period according to the metabolites eluted during this period. Metabolite analyses were performed according to the reported method (Wang et al., 2018). Based on the self-built database MWDB (Metware Biotechnology Co., Ltd., Wuhan, China), and a public database of metabolite information, MS was used for qualitative and quantitative analysis of the samples’ metabolites.

### Volatile metabolite analysis

One gram of the leaves’ powder was placed into a 20 mL head-space vial. The vials were sealed using crimp-top caps with TFE-silicone headspace septa (Agilent). Each vial was placed at 60°C for 10 min, and then a 65 μm divinylbenzene/carboxen/polydimethylsiloxane fiber (Supelco, Bellefonte, PA, United States) was exposed to the headspace of the sample for 20 min at 60°C. The volatile compound identification and quantification were carried out using an Agilent Model 7890 BGC and a 7000D mass spectrometer (Agilent), equipped with a 30 m × 0.25 mm × 1.0 μm DB-5 ms capillary column. Helium was used as the carrier gas at a flow rate of 1.0 ml/min. The injector temperature and detector temperature were 250°C and 280°C, respectively. The oven temperature was programmed at 40°C for 5 min, increased by 6°C/min to 280°C, and held for 5 min. The ionization voltage was 70 eV. Mass spectra were scanned from 30 to 350 amu. The compounds were identified by comparing the mass spectra with the MWGC database (Metware Biotechnology Co., Ltd., Wuhan, China) and a linear retention index.

### Network pharmacology and molecular docking analysis

A number of common potential active compounds from *A. argyi* and *A. indica* were screened according to the selected criteria of bioavailability (OB) ≥ 30% and oral drug-like (DL) ≥ 0.18. The PubChem database^33^ was used to determine the compound names and molecular structures. The target genes of these compounds were predicted by the TCMSP database and Universal Protein database (UniProt).^4^ We used “CG” as a keyword in the Online Mendelian Inheritance in Man database (OMIM),^5^ GeneCards database (GeneCards),^6^ and gene-disease associations database (DisGeNET)^7^ to search for CG-related targets and convert the target protein name to a gene name using the UniProt database. Subsequently, after removing repetitive targets, we acquired all CG targets. The target genes of *A. argyi* and *A. indica* against CG were obtained by the intersection of these target genes and the CG target gene using the online Wayne analysis tool. The candidate targets of *A. argyi* and *A. indica* used in CG therapy were predicted. The STRING database^8^ was used to construct a protein–protein interaction (PPI) regulation network. The minimum required interaction score was set to 0.9, and a two-plant-active ingredient compound-gene target-CG network was constructed using the Cytoscape 3.6.9 software by integrating the target genes for CG, the active ingredients in *A. argyi* and *A. indica*, and their corresponding targets. To determine functional term enrichment, the intersected target genes from *A. argyi* and *A. indica*, and CG were uploaded to the database for annotation, visualization, and integrated discovery (DAVID 6.8).^9^ Gene Ontology (GO) enrichment analysis (*p* < 0.05) and Kyoto Encyclopedia of Genes and Genomes (KEGG) pathway analysis (*p* < 0.05) were performed using the bioinformatics platform.^10^ Based on the above compound and target information, the protein structure of the corresponding target was obtained from the PDB database.^11^ The AutoDock Vina software^12^ was used for molecular docking analysis.

### Determination of active compound content

*A. argyi* and *A. indica* at three different growth stages were crushed with a high-throughput tissue grinder (Scientz-48 L). Then, 100 mg powder was extracted overnight at 4°C using 4.0 mL 70% aqueous methanol, and then subjected to ultrasonic extraction for 20 min to obtain the sample solution. Then, 1 mg of quercetin, luteolin, and kaempferol was added to a 10 mL volumetric flask, with 70% methanol constant volume to the scale line, and the mixed control solution (reference solution) was prepared. The reference and sample solutions were filtered through a 0.45 μm filter and analyzed by high-performance liquid chromatography (HPLC) (Thermo Scientific Dionex UltiMate 3000). Referring to the method of others (Zheng et al., 2012), the mobile phase components (methanol: water; 60: 40) were filtered through a 0.2 μm filter before use and were pumped from the solvent reservoir at a flow rate of 1 ml/min. The column temperature was 35°C, and the measured wavelength was 360 nm. HPLC chromatograms of the control and sample solutions are shown in Supplementary Figure 1.

### Cell culture and cytotoxicity analysis

RAW264.7 cells were obtained from the cell bank of the BeNa Culture Collection (Beijing, China) and cultured in Dulbecco’s modified Eagle medium (DMEM) supplemented with 10% fetal bovine serum, penicillin, and streptomycin (100 mg/mL). The cells were cultured at 37°C in an atmosphere of 5% CO_2_ for all experiments. Cytotoxicity was assessed using the MTT assay. RAW264.7 cells were seeded at a density of 1 × 10^4^ cells/well in 96-well plates (Corning Inc., Corning, NY, United States). After overnight incubation, the cells were treated with various concentrations (50, 100, 200, and 400 μg/mL) of *A. argyi* and *A. indica* for 24 h. After that, 10 μL MTT solution (0.5 mg/mL) was added to each well, and samples were incubated for 4 h. The resulting colored formazan crystals were dissolved in 150 μL dimethyl sulfoxide (DMSO) by horizontal shaking. The absorption values were measured at 570 nm with a Multimode Microplate Reader (Enspire, PerkinElmer, MA, United States). Cell viability was determined relative to the untreated cells in the control group.

### Assay to measure cell viability

Cell viability was assessed by the MTT assay. Briefly, RAW264.7 cells were seeded at a density of 1 × 10^4^ cells/well in 96-well plates. After overnight incubation, cells were treated with various concentrations (25, 50, and 100 μg/mL) of *A. argyi* and *A. indica* for 12 h, and then LPS (1 μg/mL) was added or not for the next 24 h. After that, 10 μL MTT solution (0.5 mg/mL) was added to each well, and the cells were incubated for 4 h. The resulting colored formazan crystals were dissolved in 150 μL DMSO by horizontal shaking. The absorption values were measured at 490 nm with the Multimode Microplate Reader. Cell viability was determined relative to the untreated cells in the control group, and then cell survival was calculated.

### Nitric oxide determination in the supernatant of RAW264.7 cells

RAW264.7 cells were seeded at a density of 5 × 10^5^ cells/well in 6-well plates (Corning Inc., Corning, NY, United States). After overnight incubation, cells were treated with various concentrations (25, 50, and 100 μg/mL) of *A. argyi* and *A. indica* for 12 h, and then LPS (1 μg/mL) was added or not for the next 24 h. After 24 h, the cell supernatant was absorbed for NO determination. The level of NO was determined by assaying the concentration of nitrite in the cell supernatant through the Griess reaction using the NO assay kit (Nanjing Jiancheng Institute of Biological Engineering), according to the manufacturer’s instructions.

### Real-time quantitative polymerase chain reaction

Total RNA was extracted from cultured RAW264.7 cells using the TRIzol reagent (Solarbio, China) and reverse-transcribed using the PrimeScript™ RT reagent kit with the gDNA Eraser (Perfect Real Time) (TaKaRa, Japan), according to the manufacturer’s instructions. Aliquots of the obtained cDNA samples were then amplified using PCR with the following schedule: 40 cycles at 95°C for 30 s, primer annealing at 95°C for 5 s, and extension at 60°C for 34 s. The specifically designed primers are listed in Table 1. All primers were tested, the fluorescence signals were recorded, and the relative values were compared to those of the control group.

**TABLE 1:** Oligonucleotide primers used in RT-PCR experiments.

Gene	Forward	Reverse
NLRP3	CTGGACCACCCCCTGCTG AGA	GGAAGAAGCCCTTGCACCC CTCA
IL-1β	CCAGGATGAGGACATGA GCA	CGGAGCCTGTAGTGCAG TTG
β-actin	CATGTACGTTGCTATCCA GGC	CTCCTTAATGTCACGCAC GAT

### Statistical analysis

Cluster analysis and principal component analysis (PCA) were carried out using R^13^ in accordance with previously described methods (Wang et al., 2016). K-means analysis and heatmap analysis based on hierarchical clustering were performed in R. The normalization method used for the K-means analysis was the unit variance scaling method, which involves standardizing the data according to the mean and standard deviation of the original data. Sub-classes were classified according to the trends in the relative content of metabolites in different samples. Metabolites with a fold change ≥ 2 or ≤ 0.5, and VIP ≥ 1 were defined as differentially accumulated metabolites between the compared samples. All experiments were repeated 3 times. The data was analyzed using GraphPad Prism 9 statistical software. The experimental results are expressed as means ± standard deviation (*SD*), and the comparison between groups was performed using the *t*-test or one-way ANOVA of variance. *P* < 0.05 was considered statistically significant.

## Results

### Pharmaphylogeny between *Artemisia argyi and Artemisia indica*

**FIGURE 2:**
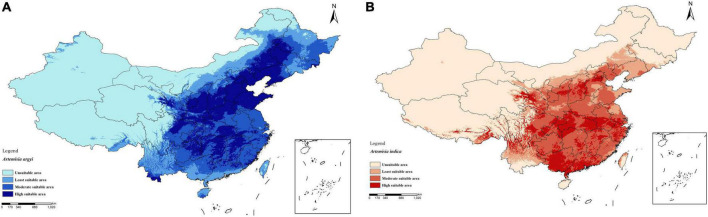
Distribution of *A. argyi* and *A. indica*. Darker color means more suitable for growth. Distribution of *A. argyi* **(A)** and *A. indica* **(B)** in China. *A. argyi* is mainly distributed in the eastern coastal areas, and *A. indica* is mainly distributed in the southeastern coastal areas.

*A. argyi* and *A. indica* are mixed for use in some areas, but the specific locations have not been recorded. Therefore, we visualized the distribution of *A. argyi* and *A. indica* in China and found these species to be distributed in almost all provinces and cities as shown in Figure 2. *A. argyi* is mainly distributed in the eastern coastal areas, and *A. indica* is mainly distributed in the southeastern coastal areas. In addition, we divided four types of regions according to the distribution of the two plants in each region. Plants are widely distributed in the “high suitability area” and less distributed in the “low suitability area.” The widespread distribution of these two plants in China explains the mixed use of *A. argyi* and *A. indica* in many areas.

**FIGURE 3:**
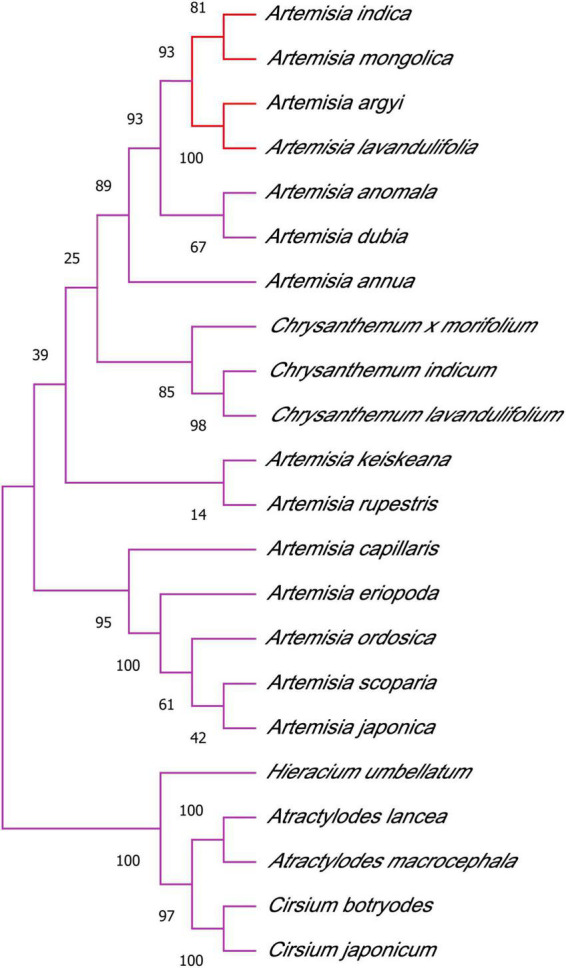
Phylogenetic tree of Asteraceae species using the neighbor-joining method. *A. argyi*, *A. indica*, *A. lavandulifolia*, and *A. mongolica* are closely related.

As shown in Figure 3, the results of the phylogenetic analysis showed that four strains, *A. argyi*, *A. indica*, *A. lavandulifolia*, and *A. mongolica*, separately formed a small branch. Thus, these species are closely phylogenetically related and can be clearly distinguished.

### Flavonoid metabolite analysis

A typical total ion content (TIC) diagram of a quality control (QC) sample is shown in Supplementary Figure 2. The TIC diagram shows a continuous description of the total intensity of all ions in the mass spectrum. The detection diagram of metabolites in the MRM mode is shown in Supplementary Figure 3, which shows the ion current diagram of various substances. Supplementary Figure 4 shows the high degree of overlap of TIC plots between multiple QC samples. The consistency of retention time (RT) and peak intensity of the two QC samples showed good signal stability when detecting the same sample at different times. High correlation coefficients (*r* = 0.989–0.998) were obtained between the three biological replicates of each sample (Supplementary Figure 5), indicating good sample uniformity. These results suggest that the data obtained in this study have good reproducibility and reliability.

The leaf samples of *A. argyi* and *A. indica* obtained at different growth stages were analyzed using LC-MS. A total of 311 flavonoids were identified, including 160 flavones (51.45%), 61 flavonols (19.61%), 21 dihydroflavones (6.75%), 17 flavonoid carbonosides (5.47%), 16 isoflavones (5.14%), 10 tannins (3.22%), eight flavanols (2.57%), eight dihydroflavonols (2.57%), seven chalcones (2.25%), two sinensetins (0.64%), and one dihydroisoflavone (0.32%). Information about the metabolite sequence number, molecular weight, formula, ionization model, metabolite name, classification, CAS number, level A or B identification confidence, CPD ID, and KEGG map is listed in Supplementary Table 1. A total of 236 compounds were identified in *A. argyi*, 216 in the early stage, 227 in the middle stage, and 230 in the later stage. A total of 214 compounds were identified in *A. indica*, 183 in the early stage, 189 in the middle stage, and 191 in the late stage.

**FIGURE 4:**
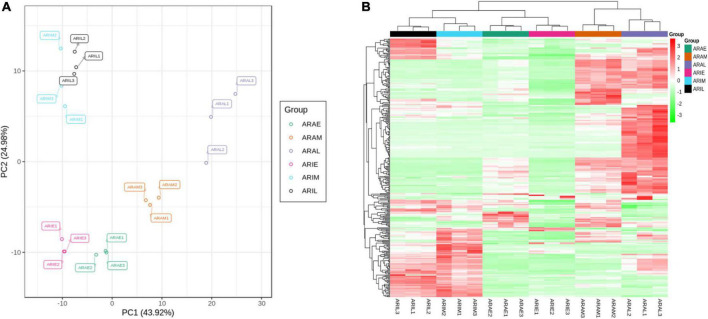
Differential flavonoid metabolites analysis. **(A)** PCA of flavonoid metabolites of *A. argyi* and *A. indica*. Each point in the figure represents a sample, and samples of the same group are represented by the same color. **(B)** Clustering heat map of flavonoid metabolites of *A. argyi* and *A. indica*. The X-axis represents the name of the sample, and the Y-axis represents the metabolite information. Different colors are the values obtained after the standardization of the relative content, in which red represents the high content and green represents the low content.

We used PCA to cluster all the flavonoid metabolites identified in the different samples. This comparison showed that there was a difference between the six groups, and all samples were in the 95% confidence interval (Hotelling’s T-squared ellipse). In the PCA score chart, the metabolic phenotypes of *A. argyi* and *A. indica* were separated, and *A. argyi* and *A. indica* could also be distinguished in each growth stage (Figure 4A). In addition, the metabolic profile differences of *A. argyi* and *A. indica* during the different growth stages were further estimated using hierarchical cluster analysis (HCA). The metabolic profile differences during the different growth stages were clearly divided into six groups on the heat map; the results showed that there were some differences in metabolite content between the two cultivars in different growth stages (Figure 4B).

**FIGURE 5:**
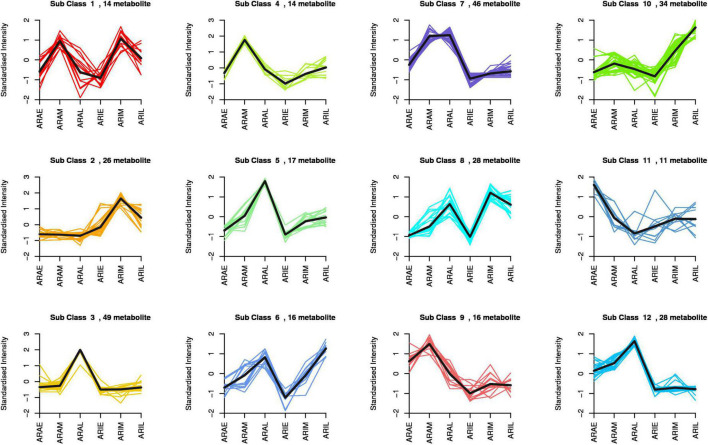
K-means analysis of differential flavonoid metabolites of *A. argyi* and *A. indica*. The X-axis represents the name of the sample, the Y-axis represents the standardized relative content of metabolites, the subclass represents the number of metabolites with the same trend.

The difference in flavonoid metabolites between *A. argyi* and *A. indica* were analyzed using a LC-MS/MS detection platform and multivariate analysis. K-means analysis divided the 299 differential flavonoid metabolites into 12 clusters (Figure 5). Among them, 94 metabolites were significantly increased in ARAL, and mainly included butin, acacetin, quercetin, pectolinarigenin, and kumatakenin. A total of 30 metabolites were significantly accumulated in ARAM. Fifty-four metabolites were significantly increased in ARIM, primarily isorhamnetin, luteolin-7-O-glucuronide, and scutellarin (Supplementary Table 2). During the same period, the metabolites of the two varieties showed different accumulations. There were 185 different metabolites in ARAE vs. ARIE (30 upregulated and 155 downregulated), 181 between ARAM and ARIM (59 upregulated and 122 downregulated), and 189 between ARAL and ARIL (55 upregulated, 134 downregulated). The trends of these different metabolites are clearly displayed in the volcano plots (Supplementary Figures 6A–C). In the same variety, there were 173 significantly different metabolites between ARIE vs. ARIL (173 upregulated, 8 downregulated, Supplementary Figure 6D), and 182 between ARAE vs. ARAL (156 upregulated, 26 downregulated, Supplementary Figure 6E). Most of the metabolites were upregulated.

### Volatile metabolite analysis

To investigate the dynamic changes in the metabolic profiles of the volatile substances, a metabolomics method was used to analyze the samples of *A. argyi* and *A. indica* at different growth stages. The mass spectrum results of QC samples were overlapped and analyzed, as shown in Supplementary Figure 7. The results showed that the TIC curve of the QC samples has a high overlap rate, which indicates that the test results are in good agreement with the experimental results.

**FIGURE 6:**
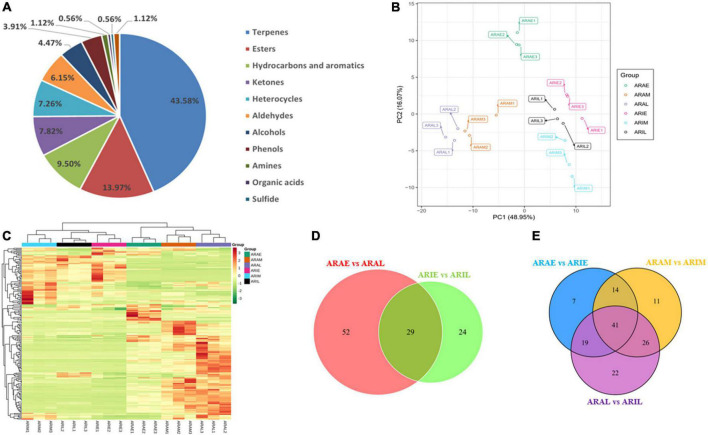
Differential volatile metabolites analysis. **(A)** Volatile compounds identified by GC-MS. **(B)** PCA of volatile metabolites. **(C)** Hierarchical cluster analysis of volatile metabolites. **(D,E)** Venn diagrams of *A. argyi* and *A. indica* in different growth stages.

A total of 179 volatile metabolites in the two varieties were identified using GC-MS/MS, including 44% terpenoids, 14.4% esters, 9% hydrocarbons and aromatics, 8% ketones, 7% heterocycles, 6.4% aldehydes, 4% alcohols, 4% phenols, 1% amines, 0.6% organic acids, 0.6% sulfides, and 1% other metabolites (Supplementary Tables 3, 4 and Figure 6A). In total, 174 compounds were obtained in *A. argyi*, including 166 in ARAE, 156 in ARAM, and 158 in ARAL; 165 compounds were identified in *A. indica*, including 144 in ARIE, 149 in ARIM, and 147 in ARIL. A total of 117 compounds were common between *A. argyi* and *A. indica*. PCA models based on 18 test samples showed differences in each group. The PCA results of the six sample groups showed significant metabolic differences among the different varieties in the different periods (Figure 6B). The accumulation pattern of metabolites in *A. argyi* and *A. indica* is directly displayed in the hierarchical cluster analysis of the heat map, which shows that the different varieties have different metabolite accumulations in different growth stages and that they may have different metabolic patterns (Figure 6C).

Based on the GC-MS/MS detection platform and multivariate analysis, the differences in volatile metabolites in *A. argyi* and *A. indica* were analyzed. A Venn diagram was used to describe the accumulation of different metabolites at different growth stages (Figures 6D,E). In the comparison between ARAE and ARAL, there were 81 differential metabolites, of which 57 were upregulated and 24 were downregulated. In the comparison between ARIE and ARIL, 53 differential metabolites were identified, of which 51 were upregulated and two were downregulated. Comparing the data of ARAE and ARIE, we identified 81 differential metabolites, of which 16 were upregulated and 65 downregulated. In the comparison between ARAM and ARIM, 92 differential metabolites were found, of which 35 were upregulated and 57 downregulated, whereas in the comparison between ARAL and ARIL, 108 differential metabolites were identified, of which 33 were upregulated and 75 downregulated. These trends are depicted in the volcano plot (Supplementary Figure 8).

### Network pharmacology analysis of *Artemisia argyi and Artemisia indica* in the treatment of chronic gastritis

Based on the above flavonoid metabolite information, the TCMSP database, and OB ≥ 30% and DL ≥ 0.18 as the screening conditions, a total of 23 active compounds from *A. argyi* and *A. indica* were identified (Table 2). These active compounds were obtained from the TCMSP database to identify the corresponding target proteins, which were converted into standard gene names using the UniProt database. After deduplication, 202 potential targets of the above compounds were identified.

**TABLE 2:** Identification of common potential active compounds from *A. argyi* and *A. indica*.

ID	MOL_ID	Compounds	Class	OB (%)	DL
A1	MOL006436	Okanin	Chalcones	98.81	0.20
A2	MOL005190	Eriodictyol	Dihydroflavones	71.79	0.24
A3	MOL002975	Butin	Dihydroflavones	69.94	0.21
A4	MOL002844	Pinocembrin	Dihydroflavones	64.72	0.18
A5	MOL004328	Naringenin	Dihydroflavones	59.29	0.21
A6	MOL004093	Azaleatin	Flavonols	54.28	0.30
A7	MOL004112	Patuletin	Flavonols	53.11	0.34
A8	MOL000239	Kumatakenin	Flavonols	50.83	0.29
A9	MOL000354	Isorhamnetin	Flavonols	49.60	0.31
A10	MOL005229	Artemetin	Flavones	49.55	0.48
A11	MOL000098	Quercetin	Flavonols	46.43	0.28
A12	MOL000422	Kaempferol	Flavonols	41.88	0.24
A13	MOL005842	Pectolinarigenin	Flavones	41.17	0.30
A14	MOL002776	Baicalin	Flavones	40.12	0.75
A15	MOL011604	Syringetin	Flavones	36.82	0.37
A16	MOL000006	Luteolin	Flavones	36.16	0.25
A17	MOL003044	Chrysoeriol	Flavones	35.85	0.27
A18	MOL001689	Acacetin	Flavones	34.97	0.24
A19	MOL004083	Tamarixetin	Flavones	32.86	0.31
A20	MOL002322	Isovitexin	Flavones	31.29	0.72
A21	MOL002881	Diosmetin	Flavones	31.14	0.27
A22	MOL001735	Hispidulin	Flavones	30.97	0.27
A23	MOL007274	Cirsimaritin	Flavones	30.35	0.30

**FIGURE 7:**
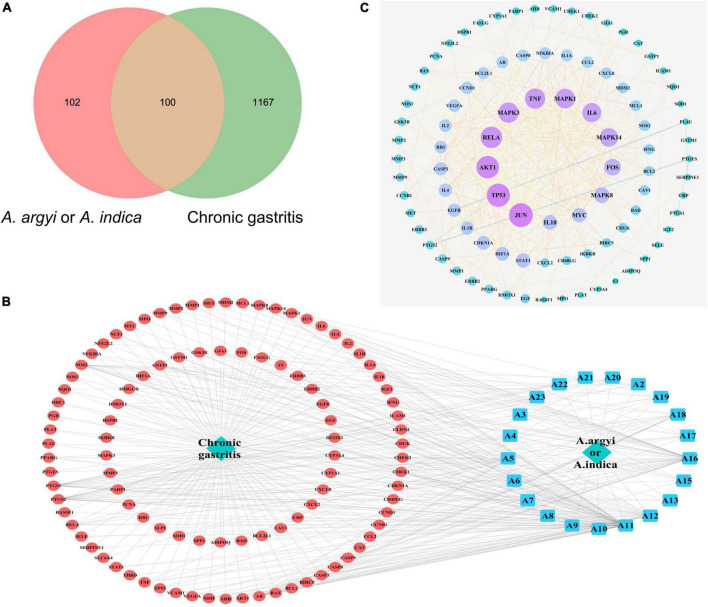
Diagram of interactions between drug targets and disease targets. **(A)** Venn diagram of the intersection of target genes of *A. argyi* and *A. indica* and chronic gastritis (CG)-related genes. **(B)** Topological network of CG target genes of *A. argyi* and *A. indica*. **(C)** Network map of *A. argyi* and *A. indica*-compound-drug action target-disease. The larger node represents the higher degree.

By integrating the CG targets collected in the OMIM database, GeneCards database, and DiGSeE database, and eliminating duplicate targets, a total of 1,267 target proteins related to CG were obtained (Supplementary Figure 9A). After matching the targets of the common active compounds with the targets of CG, the intersection was determined, leading to 100 common target genes being identified, as shown in Figure 7A.

We used Cytoscape 3.6.9 to construct the “active components-target-disease” network. The compounds and overlapped targets between *A. argyi* and *A. indica* and CG were imported into the system, and the “*A. argyi* and *A. indica* component-target-CG” network was constructed by connecting to the predicted targets. The core nodes were screened according to network topology characteristics, such as the node degree value. The results showed that quercetin, luteolin, and kaempferol play a critical role in the entire network, and that they may be significant active compounds for the treatment of CG (Figure 7B).

Based on the overlapping targets between *A. argyi* and *A. indica* and CG, a PPI network was established. There were 92 nodes and 421 edges in the network diagram, and the average node degree was 9.15 (Supplementary Figure 9B). Proteins are represented as nodes with different colors, and the lines connecting the nodes represent the functional association between proteins. The line thickness represents the confidence in the reported association. The number of connections of a node represents the degree of the node. The higher the degree, the greater the position of the gene in the regulatory network, indicating that it is a central gene. According to the topological network analysis results, the top nine core genes with a degree value greater than 20 are shown in Figure 7C. The results showed that JUN, TP53, AKT1, RELA, MAPK3, TNF, MAPK1, IL6, and MAPK14 were the most connected key targets with other genes.

**FIGURE 8:**
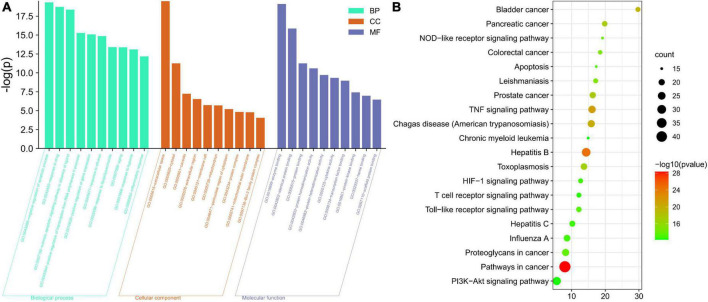
Diagram of enrichment analysis results. **(A)** Gene Ontology enrichment analysis for key targets (top 10 were listed). **(B)** KEGG pathway enrichment analysis of key targets (top 20 were listed); the abscissa label represents fold enrichment of pathways.

GO and KEGG enrichment analyses were used to analyze the anti-CG function of *A. argyi* and *A. indica*. The target genes were mostly enriched in negative regulation of apoptotic process (GO:0043066), response to drug (GO:0042493), and extrinsic apoptotic signaling pathway in absence of ligands (GO:0097192) in BP enrichment analysis, extracellular space (GO:0005615), cytosol (GO:0005829), and caveola (GO:0005901) in CC analysis, and enzyme binding (GO:0019899), identical protein binding (GO:0042802), and protein binding (GO:0005515) in MF analysis (Figure 8A). The most important pathways mediating the effects of *A. argyi* and *A. indica* on CG treatment were the NOD-like receptor, TNF, hypoxia-inducible factor (HIF)-1, T cell receptor, Toll-like receptor, and PI3K-AKT signaling pathways (Figure 8B).

**FIGURE 9:**
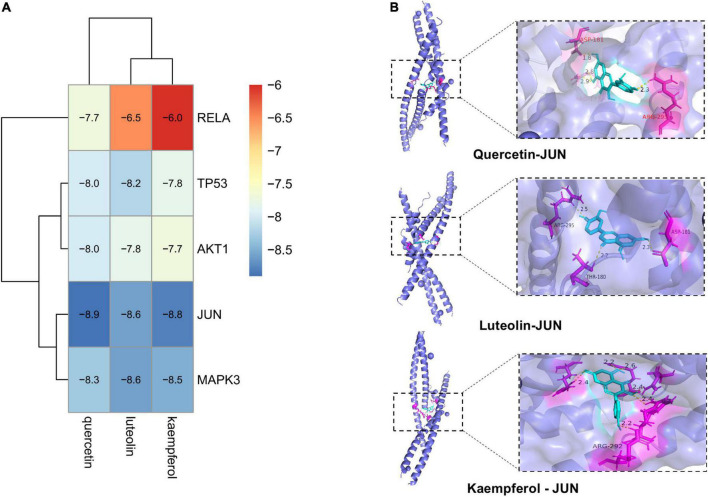
Diagram of molecular docking results. **(A)** Heat map of binding energy between the compounds of *A. argyi* and *A. indica* and core target molecule docking. The smaller the value the stronger the combination. **(B)** Docking model diagram of the compound and key target molecule.

We selected the top five targets (JUN, TP53, AKT1, RELA, and MAPK3) in the PPI network and three key compounds (quercetin, luteolin, and kaempferol) for the molecular docking analysis. Figure 9A shows a heatmap depicting the docking results, and the docking visualization results are shown in Figure 9B. The results showed that the binding energy of each compound to protein was < -5 kcal/mol, indicating that each compound could bind well to the protein. The binding energies of quercetin, luteolin, and kaempferol to JUN were -8.9, -8.6, and -8.8 kcal/mol, respectively.

### Content analysis of active compound

**FIGURE 10:**
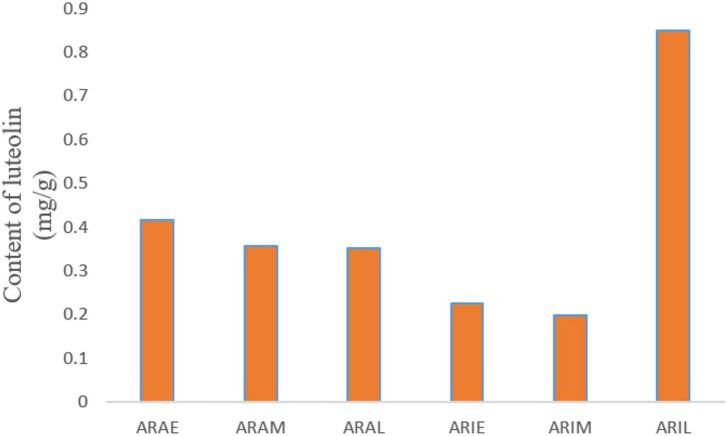
Diagram of luteolin content. X-axis is the sample name, and Y-axis is the content of luteolin in the sample powder.

The mixed reference solution was injected in the following sequence: 0.1, 0.2, 0.5, 1, 2, and 5 μL. The sample quantity (μg) was taken as the abscissa and the peak area as the ordinate, and the standard curve was drawn. The linear equation is: *y* = 66.921x -0.2352, *R*^2^ = 0.9995. The whole grass powder of *A. argyi* and *A. indica* was carefully weighed and numbered as: ARAE 100.4 mg, ARAM 100.4 mg, ARAL 100.3 mg, ARIE 100.2 mg, ARIM 100.0 mg, and ARIL 100.2 mg. There was a good linear relationship between sample quantity and peak area in the range of 0.04-0.2 mg., and the chromatographic peaks of these two compounds were not detected. The content of luteolin in the samples is shown in Figure 10. In different growth periods, the content of luteolin in *A. argyi* was the highest in April and that of *A. indica* was highest in June.

### Cytotoxicity analysis and protective effects of the extracts on RAW264.7 cells

**FIGURE 11:**
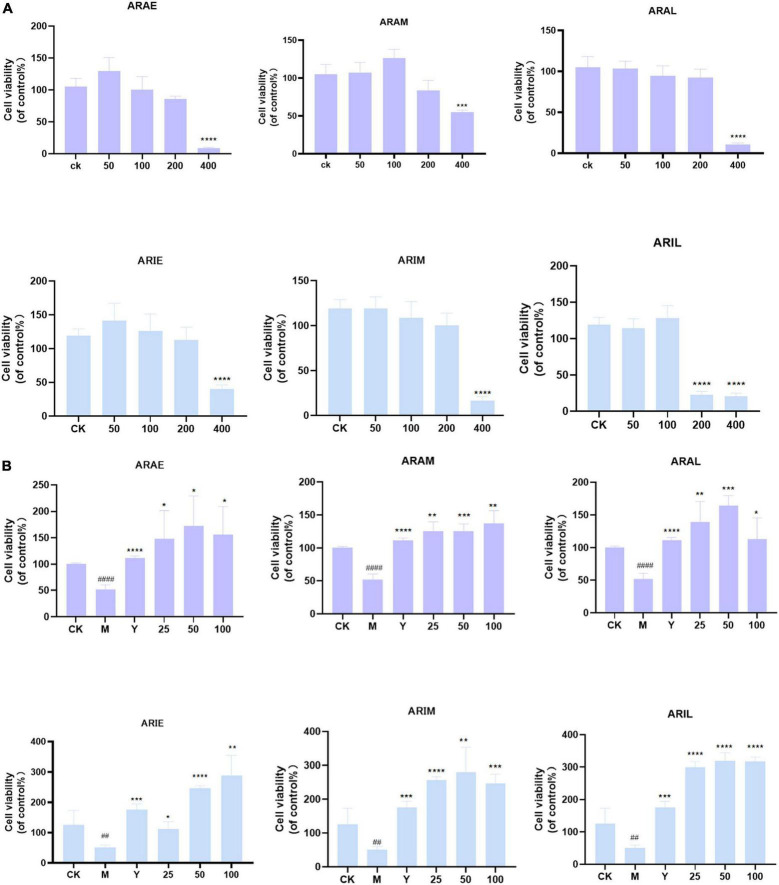
Effect of toxicity **(A)** and activity **(B)** of *A. argyi* and *A. indica* on survival rate of RAW264.7 macrophages. *A. argyi* and *A. indica* between 0 and 100 μg/ml had no toxic effect on RAW264.7 cells. The activity of ARAL was higher than that of ARAE and ARAM, and that of ARIL was higher than that of ARIE and ARIM in RAW2647 macrophages (means ± SD, *n* = *3*). ****P < 0.0001, ***P < 0.001, **P < 0.01, *P < 0.1 compared with control group. ^####^*P* < 0.0001, ^#^*P* < 0.1 compared with that of the control group.

First, we determined the effects of different doses of *A. argyi* and *A. indica* on the viability of RAW264.7 cells using the MTT assay to determine the effect of the drug on cytotoxicity. Figure 11A shows that *A. argyi* and *A. indica* had no cytotoxicity when the dosage was ≤ 100 μg/mL in all three growth stages stage. Therefore, three concentrations were selected (25, 50, and 100 μg/mL) for subsequent experiments.

As shown in Figure 11B, compared with the model group, ARAE, ARAM, and ARAL had significantly improved cell survival rate and ARAL had the best effect. Interestingly, *A. indica* was consistent with *A. argyi*, as *A. indica* harvested in June (ARIL) significantly improved cell survival compared to that of the model group.

### *Artemisia argyi* and *Artemisia indica* alleviated LPS-induced RAW264.7 cell inflammation via the NOD-like receptor Signaling pathway

Based on the cell viability results, we used ARAL and ARIL to validate the network pharmacology results. The KEGG results revealed that the inflammatory pathways that may be involved in the treatment of CG using *A. argyi* and *A. indica* include the NOD-like receptor signaling pathway, TNF signaling pathway, Toll-like receptor signaling pathway, and the PI3K-Akt signal pathway. Since the NOD-like receptor signaling pathway is closely related to CG and plays a key anti-inflammatory role, two key factors in this pathway, NLRP3 and IL-1β, were selected to verify the anti-inflammatory mechanism of *A. argyi* and *A. indica*.

**FIGURE 12:**
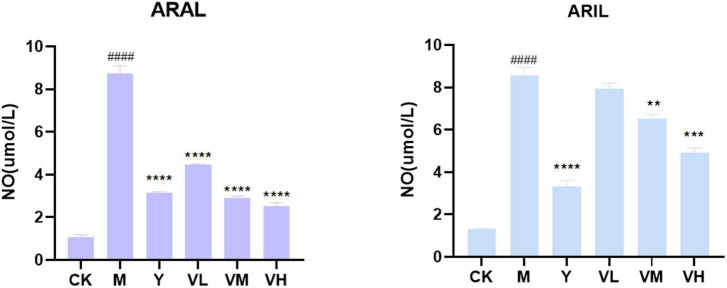
Effect of *A. argyi* and *A. indica* on NO production in RAW264.7 macrophages. The content of LPS group increased and decreased after administration (means ± *SD*, *n* = *3*). ****P < 0.0001, ***P < 0.001, **P < 0.01 compared with that of the LPS group; ^####^*P* < 0.0001 compared with that of the control group.

**FIGURE 13:**
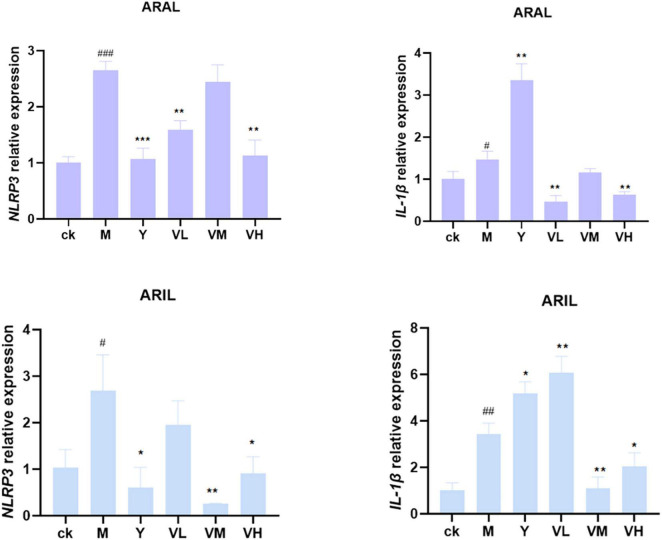
Relative expression of NLRP3 and IL-1β mRNA in RAW264.7 cells. After administration, NLRP3 and IL-1 β mRNA expression decreased (mean ± *SD*, *n* = *3*). ***P < 0.001, **P < 0.01, *P < 0.1 compared with that of the LPS group; ^###^*P* < 0.001, ^##^*P* < 0.01, ^#^*P* < 0.1 compared with that of the control group.

First, the cell supernatant was absorbed for NO determination (Figure 12); after adding LPS, the NO content was significantly increased, and by giving positive medicine (indomethacin) and different concentrations of alcohol extract of *A. argyi* and *A. indica*, the NO content decreased significantly in a dose-dependent manner (i.e., at 100 μg/ml dosage of *A. argyi* and *A. indica*, the NO content was the lowest). Based on the results of the NO kit, we then analyzed the mRNA expression of two inflammatory factors, NLRP3 and IL-1β, in RAW264.7 cells. As shown in Figure 13, compared with that in the control group, the mRNA expression of NLRP3 and IL-1β increased in the model group, and showed a significant downward trend after the administration of three concentrations of *A. argyi* extract, but it did not show a dose-dependent relationship. In addition, the results shown in Figure 13 suggest that the result of *A. indica* was basically consistent with that of *A. argyi*; compared with that in the control group, the mRNA in the model group increased and tended to decrease after *A. argyi* administration.

## Discussion

*Artemisia* is a large genus of family Asteraceae, and *A. argyi* and *A. indica* are two edible and medicinal plants with similar morphology, traditional therapeutic effects, physicochemical properties, and flowering period (see text footnote 1). In addition, *A. argyi* and *A. indica* are distributed in almost all areas of China, mostly in coastal areas, and studies have reported mixed use in some areas (Song et al., 1994; Huang and Liu, 1999; Wu et al., 2008; Wei et al., 2009; Liang et al., 2010; Qin et al., 2012). We conducted a systematic analysis of the ITS sequences of Asteraceae, and according to the results of the phylogenetic tree, *A. argyi*, *A. indica*, *A. lavandulifolia*, and *A. mongolica* are the most closely related species. A new study showed that *A. argyi* and *A. indica* are most closely related based on phylogenetic relationships constructed from chloroplast genome data of 19 species in the family Asteraceae (Lan et al., 2022). The four species belong to Sect. *Artemisia* (see text footnote 1). *A. argyi* and *A. indica* are similar in flavor, meridian tropism, and function. In pharmacophylogeny, closely related species are similar not only in morphology but also in biochemical characteristics, so their chemical compositions, including secondary plant metabolites, are often similar (Li, 2008). It has been reported that flavonoids and volatile oils are common in *Artemisia*. Metabolomics was used to analyze the changes in the contents of total flavonoids and essential oils of *A. argyi* and *A. indica*. There were 299 and 179 total flavonoids and essential oils, respectively, among which 117 essential oils were the same between *A. argyi* and *A. indica*. The total flavonoids varied in different growth stages, but there were more than 180 flavonoids similar between the two species. In addition, the content of volatile oils and flavonoids, such as eupatilin, prunetin, and artemitin, will increase with the growth of plants.

The network pharmacology results showed that both *A. argyi* and *A. indica* can treat CG. Three flavonoids in *A. argyi* and *A. indicia*, quercetin, luteolin, and kaempferol, play critical roles in the entire network, and they may be significant active compounds for the treatment of CG. Quercetin and kaempferol have previously been shown to be active ingredients in the treatment of gastric ulcers (Wang et al., 2019). Studies have shown that quercetin has good anti-inflammatory and antibacterial effects (Yao et al., 2016). It can effectively inhibit *Helicobacter pylori*, which is closely related to CG treatment. Overman et al. (2011) found that quercetin in macrophages and adipocytes can decrease the expression levels of the inflammatory genes *TNF-*α, *IL-6, IL-1*β, and *COX-2*, suppressing the activation of nuclear factor (NF-κB) and c-Jun N-terminal kinase (JNK). Luteolin, a flavonoid commonly found in medicinal plants, also has anti-inflammatory properties (Skiba et al., 2016). Wang et al. investigated the expression of inflammatory cytokines in macrophages. Luteolin can inhibit the expression of iNOS, IL-1β, IL-6, TNF-a, and CD86 (Wang et al., 2017). Kaempferol can inhibit the translocation of CagA and VacA proteins *in vitro* and downregulate pro-inflammatory cytokines (Miguel, 2009). Tang et al. found that kaempferol exerts an inhibitory effect on the activation of NF-κB and Akt in LPS plus ATP-stimulated cardiac fibroblasts and thus decreases the release of TNF-α, IL-1β, IL-6, and IL-18 (Tang et al., 2015). According to our content analysis, luteolin content was not the highest in ARAL and ARIL, which indicated that the anti-CG effect of *A. argyi* and *A. indica* is the result of multiple pathways, multiple targets, and synergistic effects of multiple compounds. The anti-CG action and mechanism of luteolin need further experimental verification.

In addition, *A. argyi* and *A. indica* and Dragon Boat Festival seem to be inseparable. In some places, people harvest *A. argyi* and *A. indica* on the Day of Dragon Boat Festival (Xue et al., 2019). Cell experiments have shown that *A. argyi* harvested in June had the strongest therapeutic effect on CG. Combined with the metabolomics and content determination results, we speculated that this might be the result of a combination of components. According to the similarities and differences between *A. argyi* and *A. indica*, we believe that these species can be used together in food. However, if used as medicine, we must pay close attention to the appropriate dosage and administration when mixing these species.

## Conclusion

To the best of our knowledge, this study was the first to explore the pharmaphylogeny of *A. argyi* and *A. indica* from multiple perspectives. The correlation between geographical distribution, traditional therapeutic effect, pharmacological action, and chemical composition of *A. argyi* and *A. indica* were analyzed. Our results clarified the reasons for the mixed use of *A. argyi* and *A. indica* in folk remedies and showed that the treatment of CG of *A. argyi* was better than that of *A. indica*, which may be the result of the accumulation of various compounds. The results provided a basis for the substitution or mixed use of *A. argyi* and *A. indica*.

## Data availability statement

The data presented in this study are deposited in the figshare repository, 10.6084/m9.figshare.19915000.

## Author contributions

ML, ZZ, and XH conceived and managed the project and reviewed the manuscript. ZC, SL, and JC conducted the experiments and wrote the manuscript. EZ, QL, BZ, CL, and ML interpreted the data. All authors have read and agreed to the published version of the manuscript.

## Footnotes

1
http://www.iplant.cn/frps

2
https://www.cvh.ac.cn/

3
https://pubchem.ncbi.nlm.nih.gov/

4
https://www.uniprot.org/

5
https://www.omim.org/

6
https://www.genecards.org/

7
http://www.disgenet.org/

8
https://string-db.org/

9
https://david.ncifcrf.gov/

10
http://www.bioinformatics.com.cn/

11
http://www.rcsb.org/

12
http://vina.scripps.edu/

13
http://www.r-project.org/

## References

[B1] Abad M. J. Bedoya L. M. Apaza L. Bermejo P. (2012). The *Artemisia* L. Genus: A review of bioactive essential oils. *Molecules* 17 2542–2566. 10.3390/molecules17032542 22388966 PMC6268508

[B2] Chen C. J. Luo D. D. Miao Y. H. Guo L. P. Liu D. H. (2021). Analysis and evaluation on leaf quality of different Artemisia argyi germplasm resources. *Chin. J. Exp. Tradit. Med. Form.* 27 129–136. 10.13422/j.cnki.syfjx.20210511

[B3] Chinese Pharmacopoeia Commission (2020). *Pharmacopoeia Of The People’s Republic Of China.* Beijing: Chinese Medical Science and Technology Press.

[B4] Editorial Board of China Bencao (1999). *China Bencao.* Shanghai: Shanghai Science and Technology Press.

[B5] Huang H. B. Liu X. C. (1999). Pharmacognostical identification of *Artemisia argyi* and *Artemisia indica*. *J. Chin. Med. Mater.* 22 283–287. 10.13863/j.issn1001-4454.1999.06.006 12575066

[B6] Lan X. Y. Zhang Y. Zhu L. B. Liu D. H. Huang X. Z. Zhou L. (2020). Research progress on chemical constituents from Artemisiae Argyi Folium and their pharmacological activities and quality control. *China J. Chin. Mater. Med.* 45 4017–4030. 10.19540/j.cnki.cjcmm.20200714.201 33164385

[B7] Lan Z. H. Tian X. F. Shi Y. H. Gao R. R. Yin Q. G. Xiang L. (2022). Chloroplast genome structure characteristics and phylogenetic analysis of *Artemisia indica*. *China J. Chin. Mater. Med* 10.19540/j.cnki.cjcmm.20220713.101 [Epub ahead of print]. 36471930

[B8] Li M. H. (2008). *A Pharmacophylogenetic Study of Salvia l. (lamiaceae) in China.* Beijing: Peking Union Medical College.

[B9] Li S. Zhou S. B. Yang W. Meng D. L. (2018). Gastro-protective effect of edible plant *Artemisia argyi* in ethanol-induced rats via normalizing inflammatory responses and oxidative stress. *J. Ethnopharmacol.* 214 207–217. 10.1016/j.jep.2017.12.023 29273436

[B10] Liang Z. L. L. K. S. Zhu Y. L. Fang F. Ye Y. Q. (2010). Study on germination characteristics of Artemisia indica Willd. seeds. *J. Anhui Agric. Sci.* 38 13034–13036. 10.13989/j.cnki.0517-6611.2010.24.087

[B11] Liu Y. K. Christer E. (2017). New specific primers for amplification of the Internal Transcribed Spacer region in Clitellata (*Annelida*). *Ecol. Evol.* 7 10421–10439. 10.1002/ece3.3212 29238565 PMC5723599

[B12] Miguel L. L. (2009). Distribution and biological activities of the flavonoid luteolin. *Mini Rev. Med. Chem.* 9 31–59. 10.2174/138955709787001712 19149659

[B13] Overman A. Chuang C. C. Mclntosh M. (2011). Quercetin attenuates inflammation in human macrophages and adipocytes exposed to macrophageconditioned media. *Int. J. Obes.* 35 1165–1172. 10.1038/ijo.2010.272 21224828

[B14] Qin W. H. Huang K. N. Huang H. X. (2012). Affect of different processing methods on flavonoids content and analgesic effect in *Artemisiae indica* from Guangxi. *Chin. J. Exp. Tradit. Med. Form.* 18 51–53. 10.13422/j.cnki.syfjx.2012.12.025

[B15] Ren Y. Y. Jiang N. P. Liu R. Y. Song L. K. Tan R. Gu J. (2017). ITS2 sequence analysis and identification of medicinal *Artemisia* plants. *China J. Chin. Mater. Med.* 42 1395–1400. 10.19540/j.cnki.cjcmm.20170222.016 29052405

[B16] Skiba M. A. Szendzielorz K. Mazur B. Krol W. (2016). The inhibitory effect of flavonoids on interleukin-8 release by human gastric adenocarcinoma (AGS) cells infected with cag PAI (+) *Helicobacter* pylori. *Cent. Eur. J. Immunol.* 41 229–235. 10.5114/ceji.2016.63119 27833438 PMC5099377

[B17] Song P. S. Zhang B. C. Wei Y. L. Liu H. F. (1994). Investigation and resource distribution of original plants of Gansu Artemisia argyi medicinal materials. *J. Chin. Med. Mater* 17 15–16. 10.13863/j.issn1001-4454.1994.09.006

[B18] Tang X. L. Liu J. X. Dong W. Li P. Li L. Hou J. C. (2015). Protective effect of kaempferol on LPS plus ATP-induced inflammatory response in cardiac fibroblasts. *Inflammation* 38 94–101. 10.1007/s10753-014-0011-2 25189464

[B19] Wang A. M. Li R. S. Ren L. Gao X. L. Zhang Y. G. Ma Z. M. (2018). A comparative metabolomics study of flavonoids in sweet potato with different flesh colors (Ipomoea batatas (L.) Lam). *Food Chem.* 260 124–134. 10.1016/j.foodchem.2018.03.125 29699652

[B20] Wang S. C. Tu H. Wan J. Chen W. Liu X. Q. Luo J. (2016). Spatio-temporal distribution and natural variation of metabolites in citrus fruits. *Food Chem.* 199 8–17. 10.1016/j.foodchem.2015.11.113 26775938

[B21] Wang S. Cao M. Xu S. Zhang J. Wang Z. Mao X. (2017). Effect of luteolin on inflammatory responses in RAW264.7 macrophages activated with LPS and IFN-γ. *J. Funct. Foods* 32 123–130. 10.1016/j.jff.2017.02.018

[B22] Wang Y. Sun Y. W. Wang Y. M. Ju Y. Meng D. L. (2019). Virtual screening of active compounds from *Artemisia argyi* and potential targets against gastric ulcer based on Network pharmacology. *Bioorg. Chem.* 88:102924. 10.1016/j.bioorg.2019.102924 31005783

[B23] Wei Z. Y. Wu H. E. Liang H. Y. (2009). Analysis of chemical constituents of volatile oil from *Artemisia indica* of Guangxi by GC–MS. *Chin. J. Ethnomedicine Ethnopharmacy* 18 27–29.

[B24] Wu H. E. Li Y. H. Wei Z. Y. Liang H. Y. (2008). A comparative study on the chemical constituents of volatile oil from *Artemisia indica*, *Artemisia feddei* and *Artemisia argyi* in Guangxi province. *China Med. Herald* 5 23–26.

[B25] Xue Z. Q. Guo L. X. Guo M. Yang G. Y. Zhang D. Guo L. (2019). Study on difference of chemical constituents of Qiai in different harvest periods. *China J. Chin. Mater. Med.* 44 5433–5440. 10.19540/j.cnki.cjcmm.20190830.202 32237391

[B26] Yang M. T. Kuo T. F. Chung K. F. Liang Y. C. Yang C. W. Lin C. Y. (2020). Authentication, phytochemical characterization and anti-bacterial activity of two *Artemisia* species. *Food Chem.* 333:127458. 10.1016/j.foodchem.2020.127458 32673952

[B27] Yao L. Yao J. Y. Han C. Y. Yang J. X. Chaudhry M. T. Wang S. N. (2016). Quercetin. Inflammation and Immunity. *Nutrients* 8:167. 10.3390/nu8030167 26999194 PMC4808895

[B28] Zhang Y. Kang L. P. Guo L. P. Zhou A. K. (2017). Herbalogical study of Mugwort leaves and research advances in its application. *Shanghai J. Acupunct. Moxibustion* 36 245–255. 10.13460/j.issn.1005-0957.2017.03.0245

[B29] Zheng G. H. Xu Y. Geng C. Jiang Y. Z. (2012). Quantification of effective ingredients in the *Artemisia sacrorum* Ledeb by reverse phase-high performance liquid chromatography. *J. Med. Sci. Yanbian Univ.* 35 113–116. 10.16068/j.1000-1824.2012.02.003

[B30] Zhou B. C. Liu Y. B. G. Y. Bi Y. Q. Xiao H. Li M. H. (2022). Mongolian medicine “Bashaga” variety systematization based on pharmacognosy and pharmacophylogeny. *Mod. Chin. Med.* [Epub ahead of print].

