# Supplementary material for: The pharmacophylogenetic relationships of two edible medicinal plants in the genus Artemisia
Source: Front Plant Sci. 2022 Aug 18;13:949743. doi: 10.3389/fpls.2022.949743 (PMC9436419; doi:10.3389/fpls.2022.949743)
Supplement: Supplementary file 1 [file Data_Sheet_1.docx]

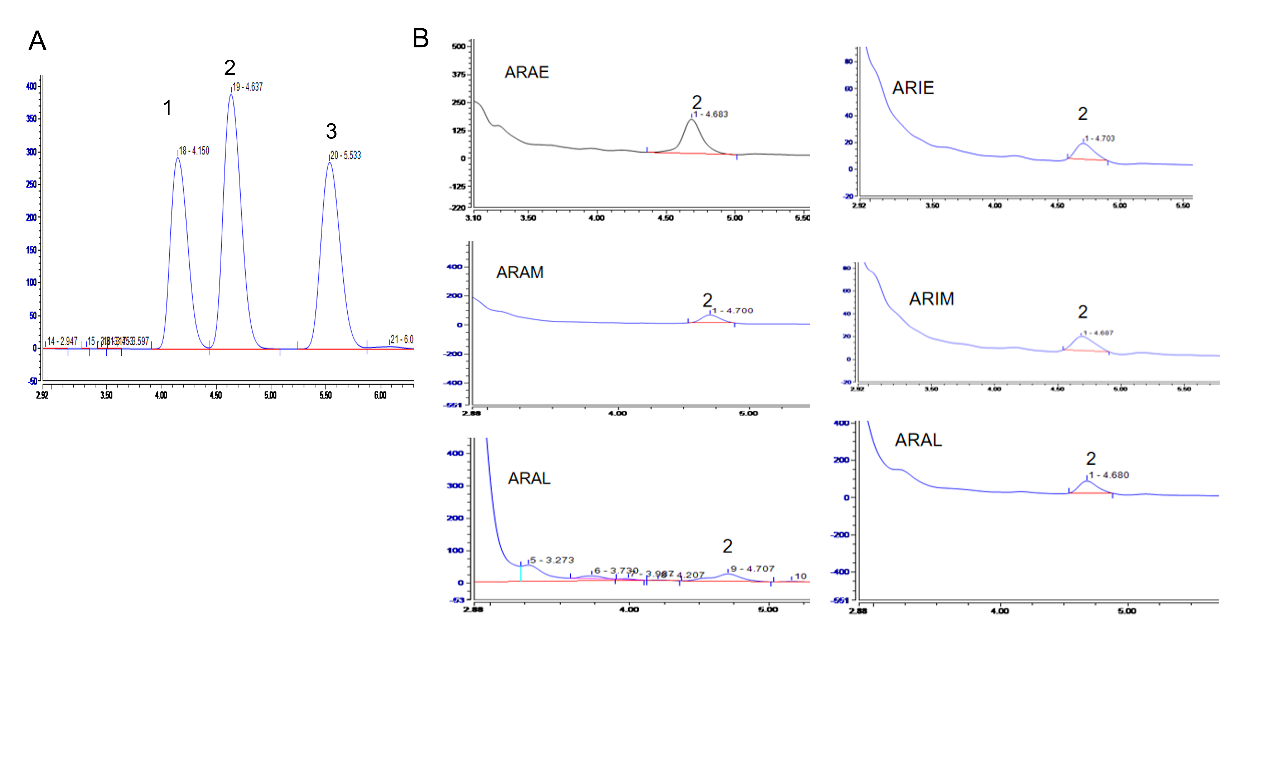


**Figure S1**. HPLC chromatograms of mixed control solution (A) and sample solution (B). 1: quercetin, 2: luteolin, 3: kaempferol.


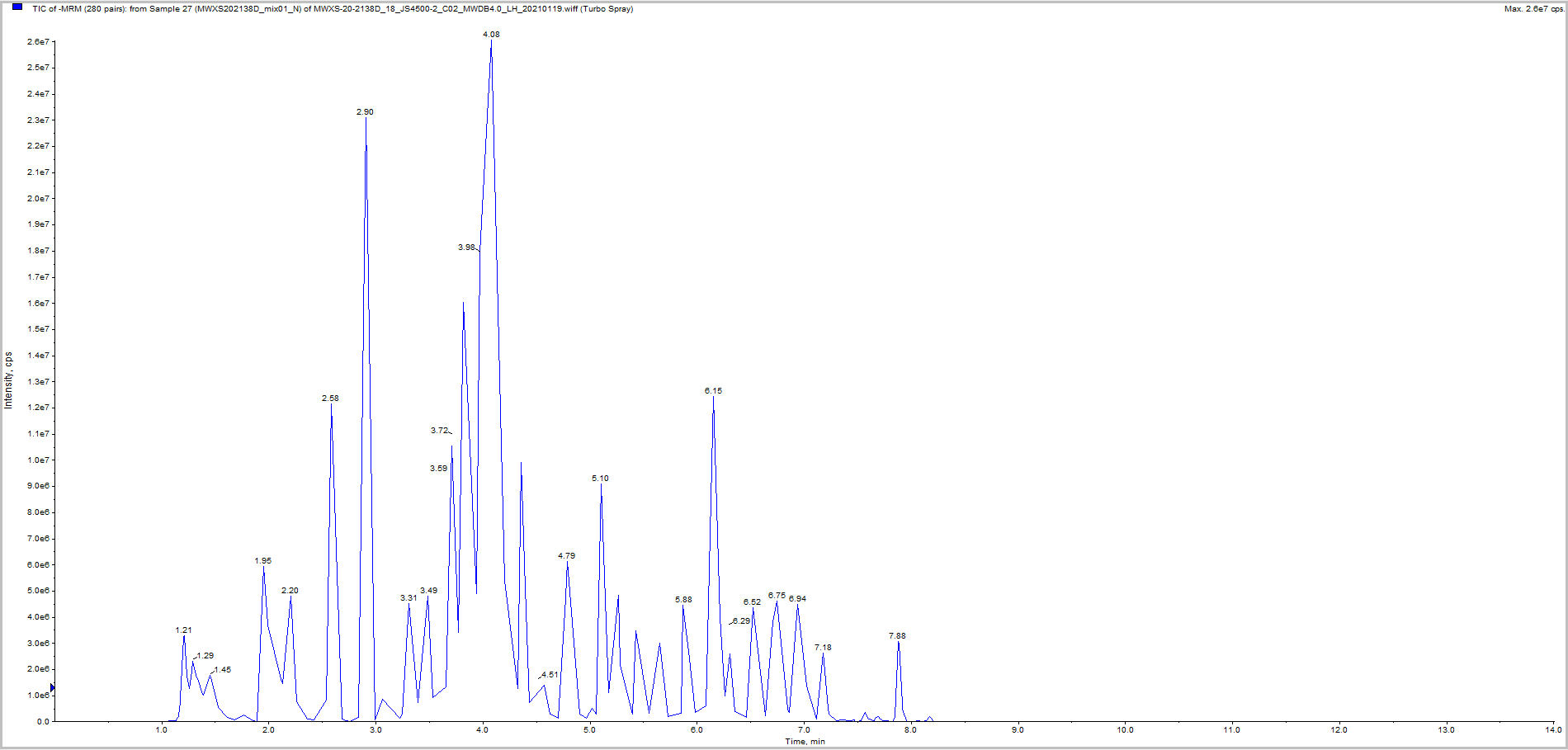

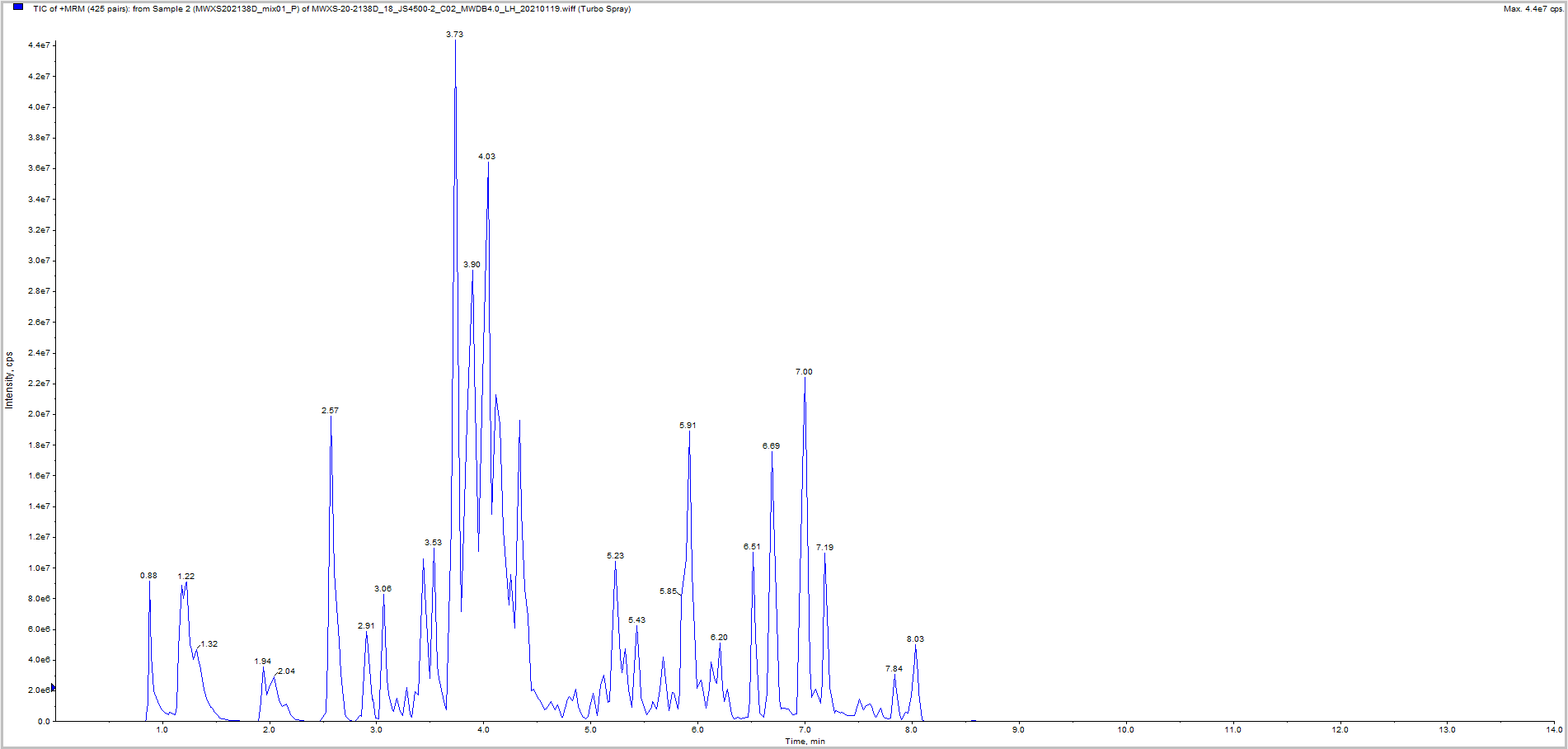


**Figure S2**. TIC diagrams of QC sample UPLC-MS.


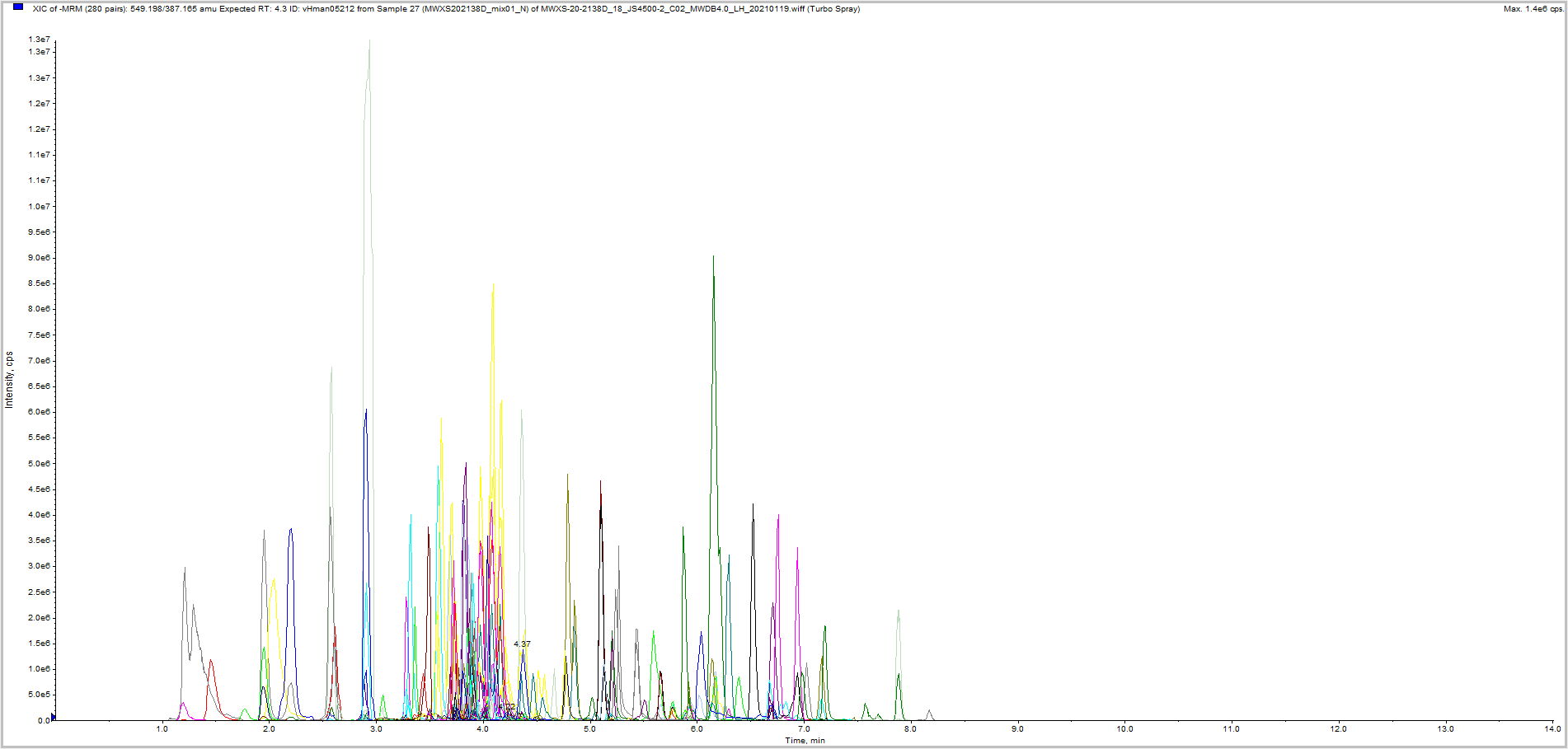

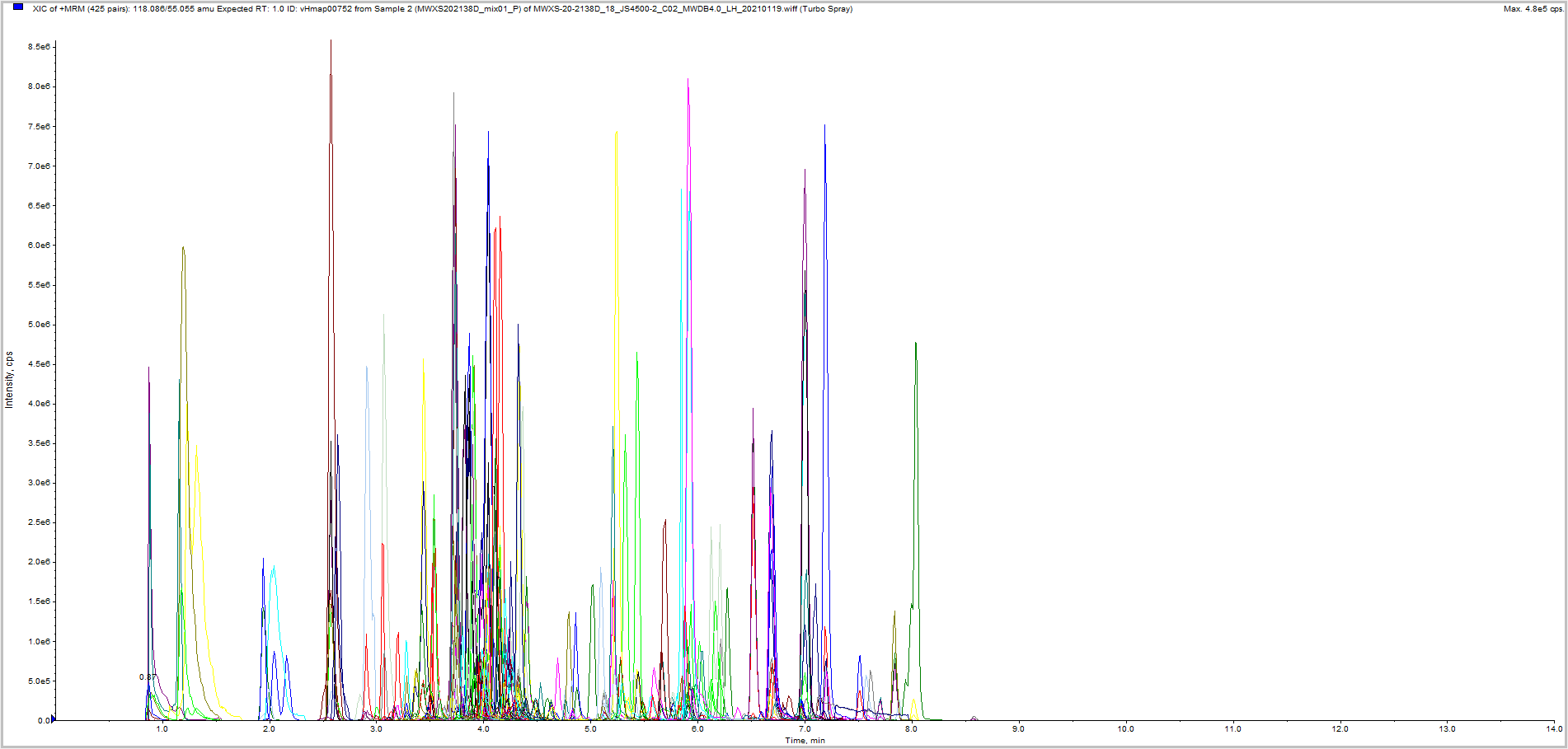


**Figure S3**. The multi peak detection diagram of metabolites in MRM mode. The abscissa represents the retention time of metabolites, and the ordinate represents the ion current intensity, in counts per second (cps). In the graph, each peak with different color represents the detected metabolite.


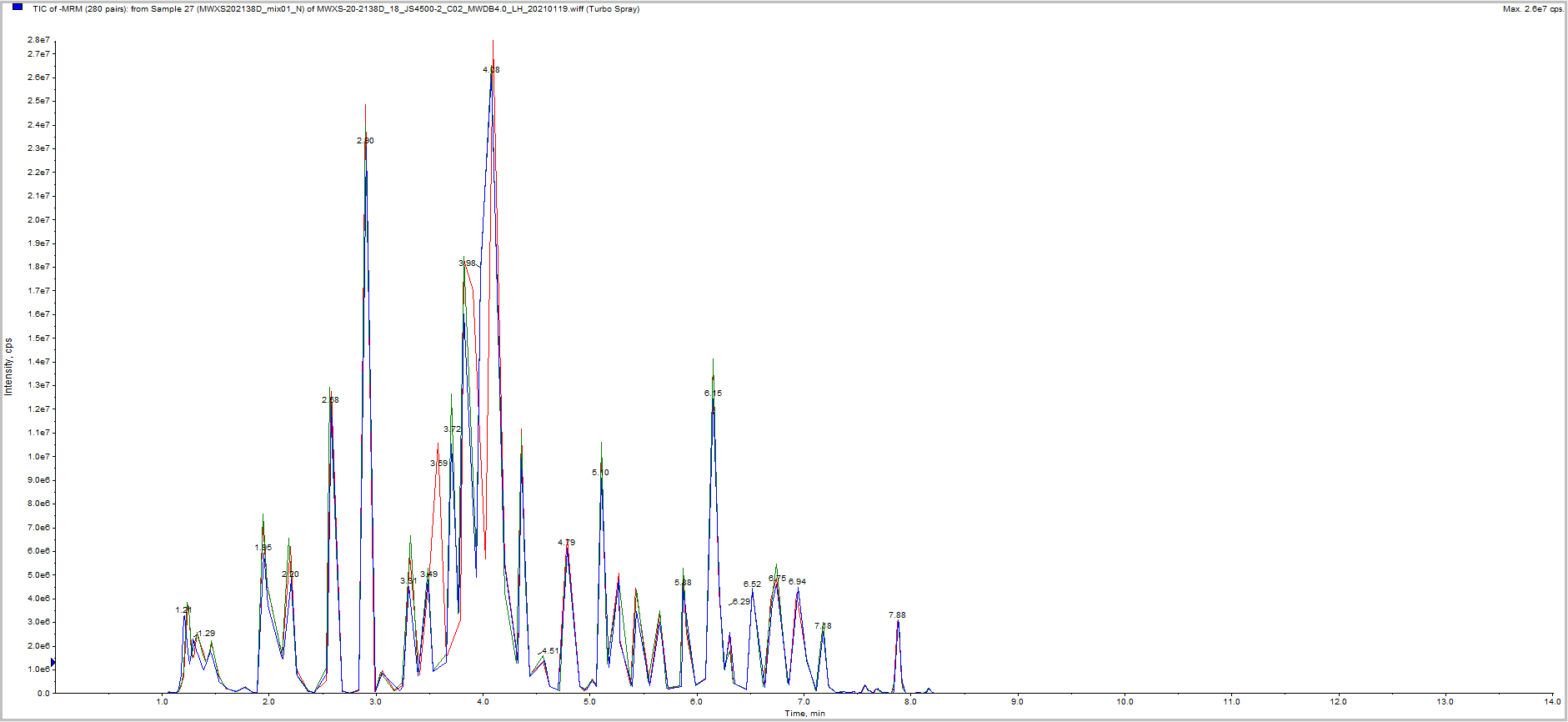

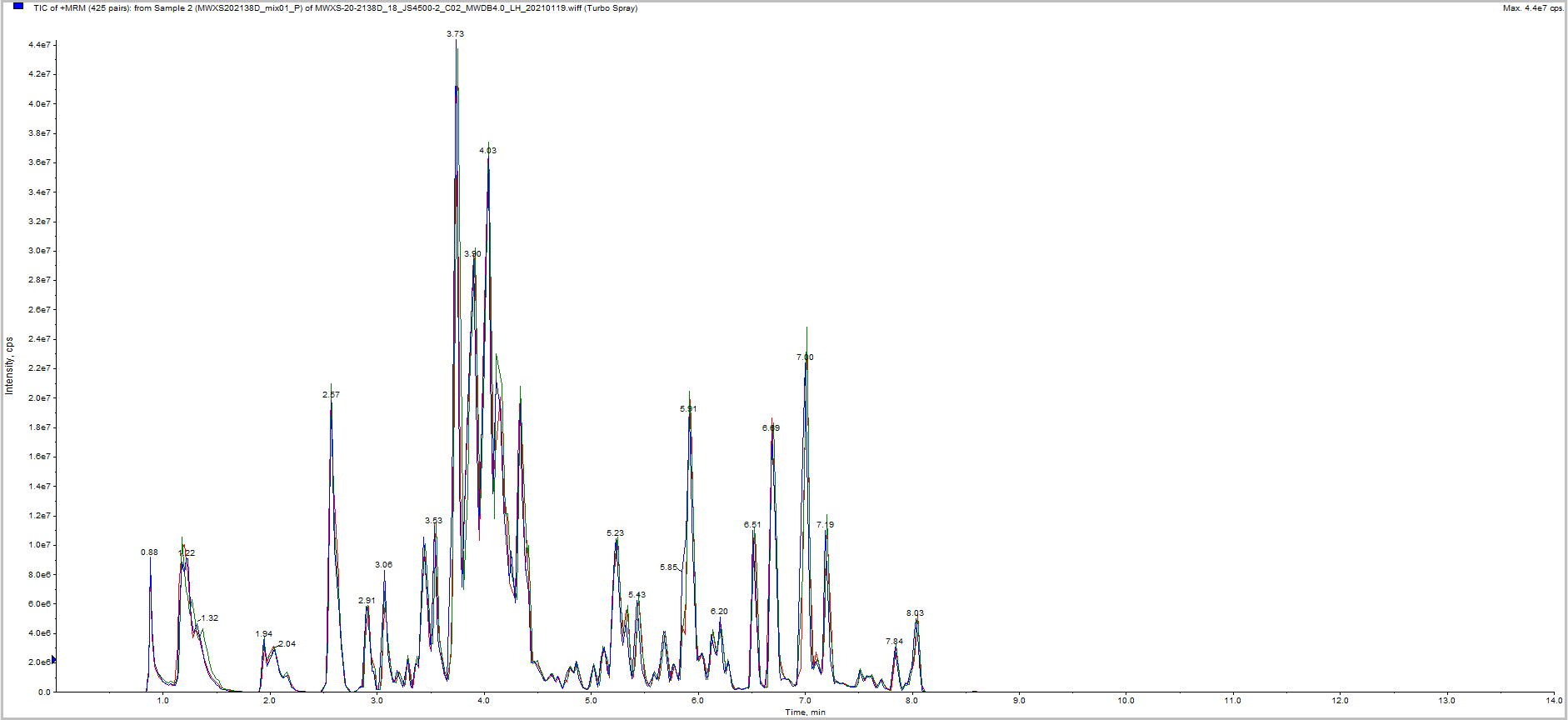


**Figure S4**. The overlap of TIC plots between multiple QC samples by UPLC-MS. (The consistency of RT and peak intensity of the two QC samples shows that it has good signal stability when detecting the same sample at different times.)


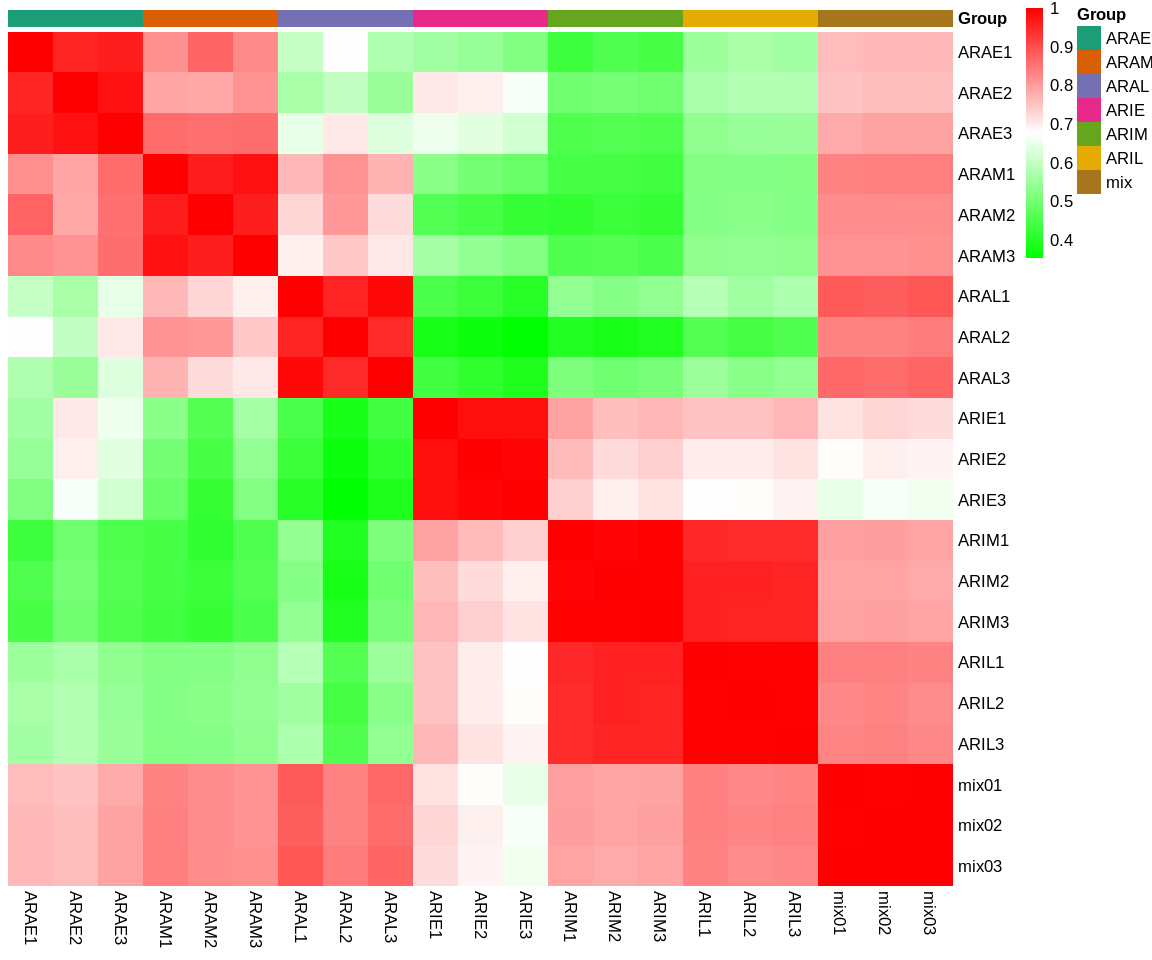


**Figure S5**. Correlation diagram between *A. argyi* and *A. indica* samples. The abscissa represents the sample name, the ordinate represents the corresponding sample name, and the color represents the correlation value. (The high correlation coefficients (r = 0.989–0.998) were obtained between the three biological replicates of each sample, indicating good sample uniformity.)


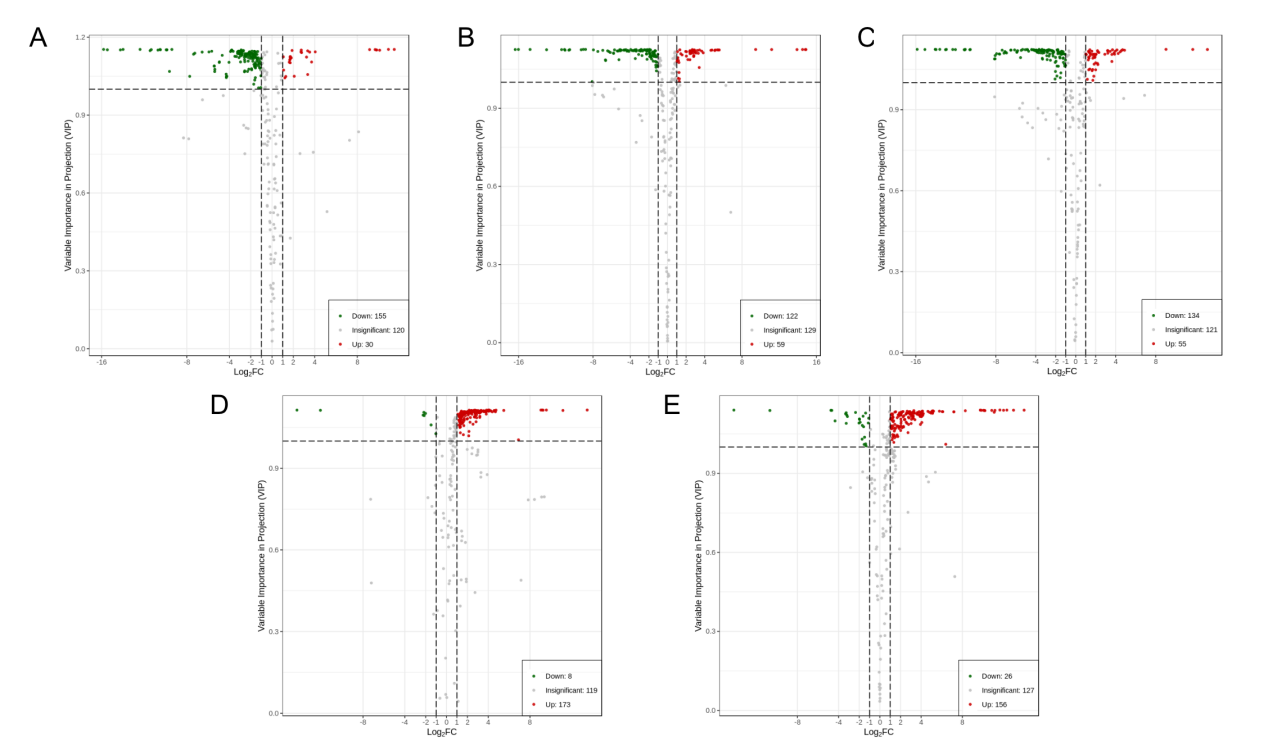


**Figure S6**. Differential flavonoid metabolites between different groups. A: ARAE vs. ARIE. B: ARAM vs. ARIM. C: ARAL vs. ARIL. D: ARIE vs. ARIL. E: ARAE vs. ARAL. Differential metabolites were defined as metabolites with fold change ≥2 (up-regulated) or ≤0.5 (down-regulated) in *A. argyi* and *A. indica.* A threshold of VIP ≥ 1was used to separate differential metabolites from insignificant metabolites.


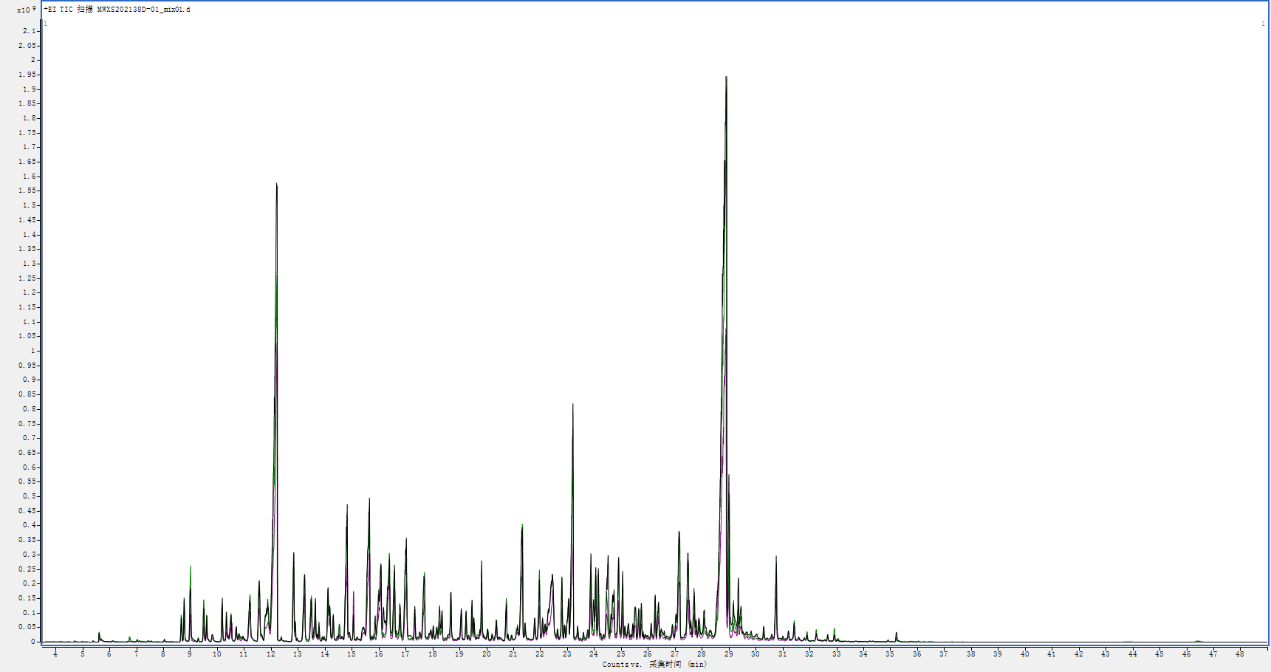


**Figure S7**. Detection of TIC overlap map of QC samples by GC-MS.（The results showed that the TIC curve of the QC samples has a high overlap rate, which indicates that the test results are in good agreement with the experimental results.）


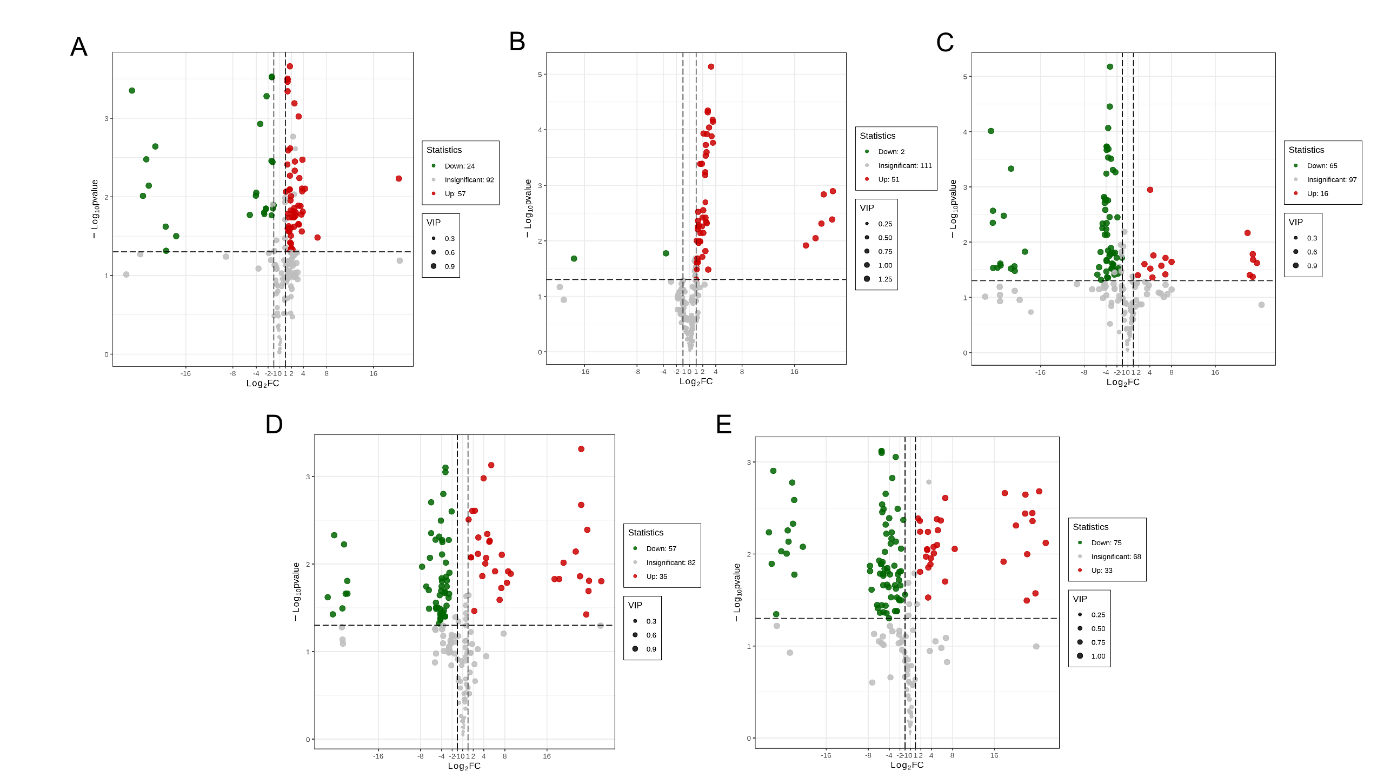


**Figure S8**. Differential volatile metabolites between different groups. A: ARAE vs. ARAL. B: ARIE vs. ARIL. C: ARAE vs. ARIE. D: ARAM vs. ARIM. E: ARAL vs. ARIL. Differential metabolites were defined as metabolites with fold change ≥2 (up-regulated) or ≤0.5 (down-regulated) in *A. argyi* and *A. indica*. A threshold of VIP ≥ 1was used to separate differential metabolites from insignificant metabolites.


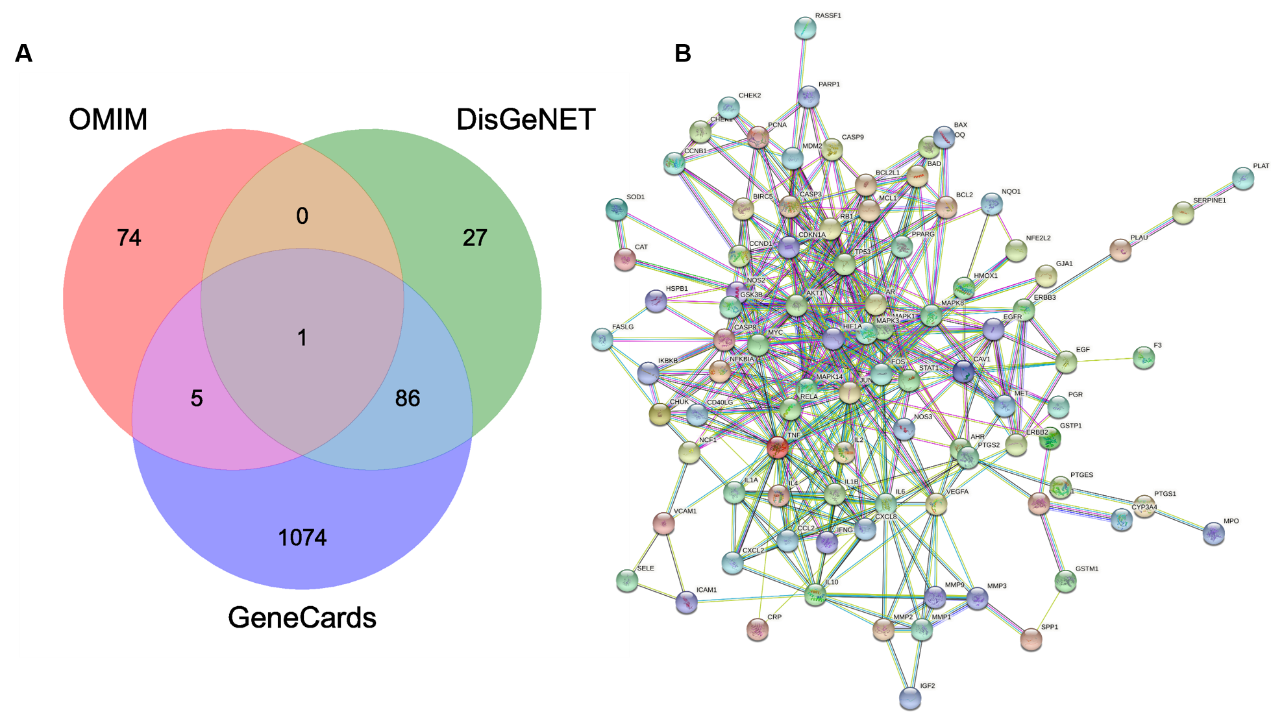


**Figure S9**. Chronic gastritis-related genes and chronic gastritis target genes. A: The Venn diagram of the intersection of chronic gastritis-related genes obtained from three databases. B: Diagram of protein interaction network of chronic gastritis target genes acted by two *Artemisia* plants.
